# Splenic PD-L1^+^ Myeloid-Derived Suppressor Cells Induce Contact-Dependent Alveolar Macrophage Dysfunction in Early Sepsis

**DOI:** 10.34133/research.1407

**Published:** 2026-08-19

**Authors:** Bing Xie, Mengqi Han, Xiaoyue Wen, Yin Yuan, Hong Qi, Yujing Zhang, Xin Wang, Zifan Zhen, Mengyuan Wang, Xinyu Zhang, Tingting Jin, Xueqiang Sun, Zhigang Wang, Xiaofeng Li, Shiying Yuan, Jiancheng Zhang, You Shang

**Affiliations:** ^1^ Department of Critical Care Medicine, Union Hospital, Tongji Medical College, Huazhong University of Science and Technology, Wuhan 430022, China.; ^2^ Key Laboratory of Anesthesiology and Resuscitation (Huazhong University of Science and Technology), Ministry of Education, Wuhan, China.; ^3^ Hubei JiangXia Laboratory, Wuhan, Hubei 430200, China.; ^4^ Hubei Key Laboratory of Regenerative Medicine and Multi-disciplinary Translational Research (Huazhong University of Science and Technology), Wuhan, Hubei 430022, China.

## Abstract

Sepsis frequently leaves patients with persistent immune impairment that contributes to late mortality, yet the anatomical routes that transmit this response to the lung remain poorly defined. Here, we identify the spleen as a key extramedullary hub orchestrating pulmonary immune paralysis through a spleen–lung axis. Mechanistically, splenic programmed death-ligand 1 (PD-L1)^+^ polymorphonuclear myeloid-derived suppressor cells undergo TFDP1-driven expansion and migrate to the lungs via CXCL2/CXCR2 signaling, establishing an interorgan immunosuppressive circuit. In the lung, these cells reprogram alveolar macrophages through programmed death-1 (PD-1)-dependent checkpoint signaling, inducing a dysfunctional state characterized by impaired antibacterial responses and enrichment of complement and immune checkpoint pathways. Genetic or pharmacologic disruption of the PD-L1–PD-1 axis restores macrophage function and improves pulmonary host defense in experimental sepsis. Together, these findings define a mechanistic link between splenic myelopoiesis and distal lung immune paralysis.

## Introduction

Sepsis is among the leading causes of global mortality and remains one of the most common life-threatening syndromes managed in intensive care units. Although early resuscitation and antimicrobial therapies have improved short-term survival, many patients die weeks to months after the initial septic episode [1]. Importantly, respiratory complications account for a substantial proportion of these deaths. The lung is both a primary target organ and a frequent site of secondary infection in sepsis, and sepsis-associated pneumonia and respiratory failure remain major clinical challenges in critically ill patients [2,3].

Emerging evidence reveals a paradox: even at the height of the inflammatory surge, immune suppression is already underway [4]. This immunosuppressive state is clinically relevant, as it predisposes patients to secondary infections, particularly in the lower respiratory tract, even after apparent stabilization of systemic inflammation [5,6]. From a pulmonary perspective, this paradox helps explain why patients with sepsis frequently develop hospital-acquired or ventilator-associated pneumonia despite appropriate antimicrobial therapy. However, the cellular and spatial mechanisms that drive early pulmonary immunoparalysis remain incompletely understood.

Rapid expansion of myeloid-derived suppressor cells (MDSCs) is a characteristic feature of the septic immune response and is thought to contribute to the shift toward immunosuppression [7,8]. Polymorphonuclear myeloid-derived suppressor cells (PMN-MDSCs) are identified in pathological settings through the integration of cellular phenotype, disease context, and experimentally demonstrated immunosuppressive activity, rather than by a single lineage-exclusive marker [9]. In the present study, murine PMN-MDSCs were phenotypically identified as viable CD45^+^ CD11b^+^ Ly6G^high^ Ly6C^low^ cells, with their suppressive activity directly validated by inhibition of T-cell proliferation and further supported by increased Arg-1, reactive oxygen species (ROS), and nitric oxide (NO). Emergency myelopoiesis is usually attributed to the bone marrow, but the spleen is increasingly recognized as an additional reservoir and production site for suppressive myeloid cells. In cancer, splenic remodeling generates a myeloid pool capable of supporting disease progression and treatment resistance [10]. This raises the intriguing possibility that in sepsis, the spleen may play a similarly active role in dictating distal organ dysfunction. In infectious contexts, immune cells from secondary lymphoid structures may migrate to distal inflamed sites [11,12]. Our previous studies have shown that immune cells can undergo directed interorgan trafficking during the acute phase of sepsis, guided by chemokine signaling [13]. Yet, the precise origins and dynamic behavior of pulmonary MDSCs in the aftermath of sepsis remain poorly understood, particularly whether they arise from extrapulmonary reservoirs and contribute to immune suppression in the lung.

One key mechanism by which MDSCs exert their suppressive effects is through overexpression of programmed death-ligand 1 (PD-L1), which interacts with programmed death-1 (PD-1) receptors on other immune cells to dampen immune activity [14–17]. While PD-1 signaling in T cells has been extensively studied [18–22], its role in macrophages is less understood. Recent evidence suggests that PD-1 expression in macrophages impairs both bacterial killing and efferocytosis [23], hinting at a broader role in immune reprogramming during sepsis. Among lung-resident macrophages, alveolar macrophages (AMs) are long-lived cells of embryonic origin and are especially vulnerable to sepsis-induced dysfunction [24–26]. These cells are key to maintaining pulmonary homeostasis and adapting to pathogens and inflammatory stress [27]. Both clinical and experimental evidence indicate that AM dysfunction is a defining feature of sepsis-associated pulmonary immunosuppression, yet the upstream signals driving this process remain unclear.

Here, we map the spatial dynamics of early sepsis-associated immunosuppression and identify a novel spleen–lung myeloid pathway. Moving beyond traditional bone marrow-centric models, we demonstrate that the spleen serves as the indispensable early extramedullary hub for MDSC emergency myelopoiesis. Specifically, we show that PD-L1^+^ PMN-MDSCs expand locally in the spleen through a TFDP1-related program, migrate to the lung in response to CXCL2, and induce strict contact-dependent AM dysfunction. These findings shift the interpretation of pulmonary immunoparalysis from an isolated lung-intrinsic process toward a systemic organ-crosstalk event, with the spleen serving as an upstream source of suppressive myeloid input.

## Results

### Splenic PD-L1^+^ PMN-MDSCs migrate to the lungs in early sepsis

**Fig. 1.**
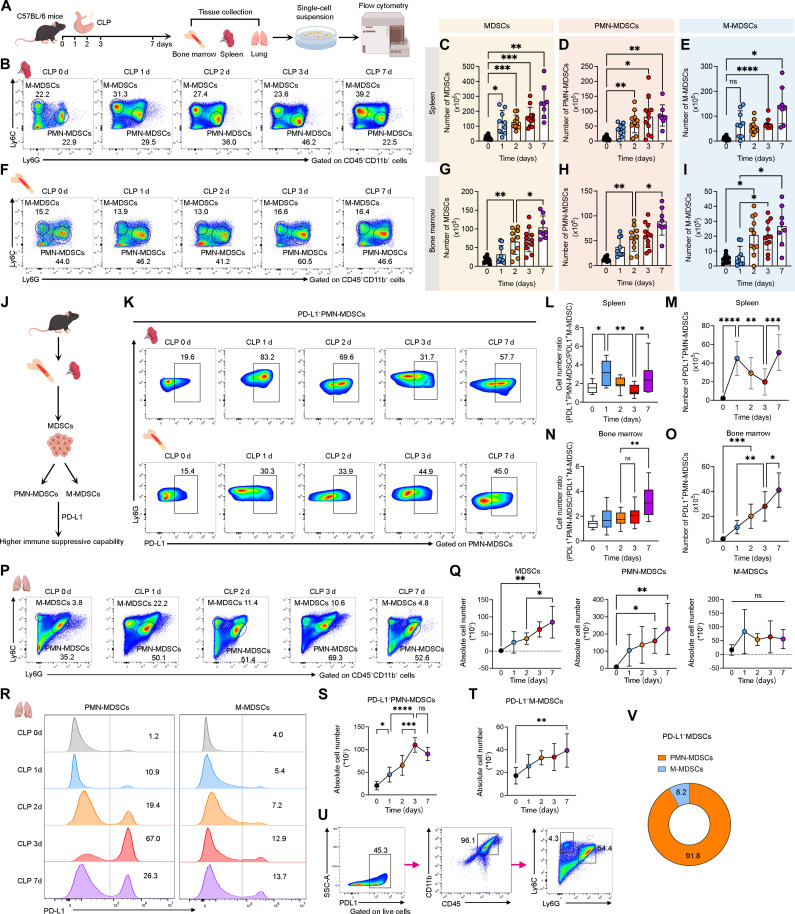
Dynamics of MDSC subsets in spleen, bone marrow, and lung during sepsis progression. (A) Schematic of CLP model and tissue collection. (B to I) Example flow cytometry (FCM) plots (B and F) with quantification of total MDSCs, PMN-MDSCs, and M-MDSCs in spleen and bone marrow from CLP mice (*n* = 8 to 11) (C to E and G to I). (J and K) Schematics (J) and representative FCM plots (K) of PD-L1^+^ PMN-MDSCs in the spleen and bone marrow. (L to O) Ratios of PD-L1^+^ PMN-MDSCs to PD-L1^+^ M-MDSCs (L and N), and kinetics of PD-L1^+^ PMN-MDSCs in spleen and bone marrow over 7 d post-CLP (*n* = 9 to 12) (M and O). (P and Q) Representative FCM plots (P) and quantification (Q) of MDSCs and their subsets in lung (*n* = 6 to 7). (R to T) Representative FCM histograms (R) and PD-L1 expression in lung PMN-MDSCs and M-MDSCs (S and T) (*n* = 6 to 7). (U and V) Representative FCM plots (U) and percentages of PMN-MDSCs and M-MDSCs among PD-L1^+^ MDSCs in lung (V). Data were plotted as mean ± SD for each data point; statistical significances were determined by one-way analysis of variance (ANOVA) followed by Tukey’s multiple-comparison test. *P < 0.05, **P < 0.01, ***P < 0.001, ****P < 0.0001. ns, not significant.

To characterize the temporal dynamics of MDSCs during sepsis, we profiled PMN-MDSC and M-MDSC subsets in the bone marrow, spleen, and lungs of mice over 7 d post-cecal ligation and puncture (CLP) (Fig. 1A). Both subsets progressively accumulated in the bone marrow and spleen from day 0 to day 7 (Fig. 1B to I). Since MDSC accumulation and activation underlie their immunosuppressive function in sepsis [28], we next assessed expression of PD-L1, a key immune checkpoint molecule associated with MDSC-mediated suppression (Fig. 1J) [14]. PD-L1 was predominantly expressed on PMN-MDSCs in both bone marrow and spleen (Fig. 1K, L, and N). Notably, in the spleen, the proportion of PD-L1^+^ PMN-MDSCs peaked sharply at day 1 post-CLP and declined markedly by day 3 (Fig. 1M), suggesting a transient wave of immunosuppression or potential migration to other sites. In contrast, PD-L1^+^ PMN-MDSCs in bone marrow showed a sustained increase over time (Fig. 1O), indicating persistent immunosuppressive activation.

**Fig. 2.**
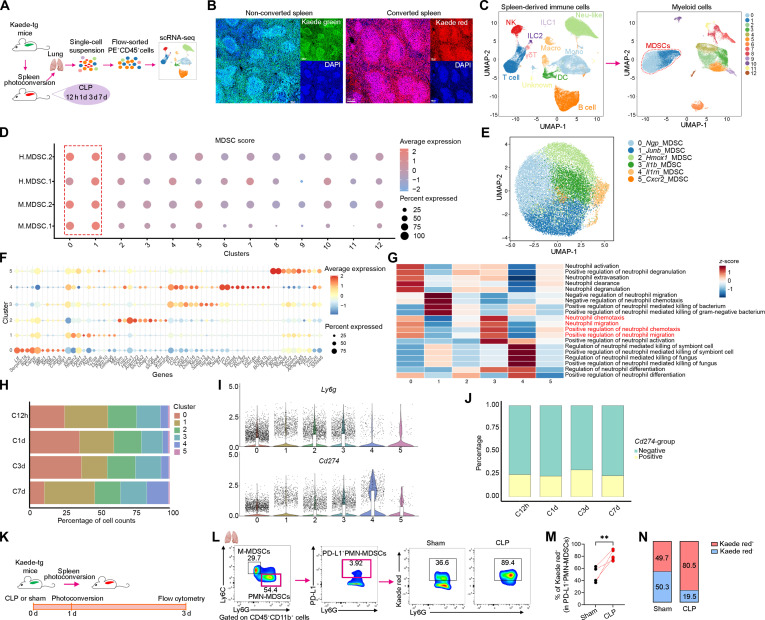
Migration of splenic PD-L1^+^ PMN-MDSCs to lung post-CLP. (A) Experimental schematic for tracking splenic-derived cells in Kaede-tg mice. Following CLP and splenic photoconversion, Kaede-red^+^ CD45^+^ immune cells were sorted from lung tissues for scRNA-seq analysis at indicated time points. (B) Representative fluorescence images demonstrating efficient photoconversion of splenic cells from Kaede-green to Kaede-red fluorescence. Scale bar, 200 μm. (C) Integrated UMAP of spleen-derived immune cells and myeloid cells from all groups. (D) MDSC signature scores across the reclustered myeloid compartment, showing enrichment of established MDSC-associated transcriptional programs in clusters 0 and 1. (E) UMAP visualization of the reclustered MDSC populations. (F) Dot plot showing the top 10 marker genes for each MDSC subcluster. (G) Enrichment of neutrophil-associated signaling pathways (from GO and Reactome databases) in MDSC subclusters. (H) The frequency of each cell type across different time points post-CLP is depicted in the columns. (I) Violin plots displaying the expression levels of *Ly6g*, a granulocytic-lineage marker, and *Cd274* (PD-L1) across all PMN-MDSC subclusters. (J) Stacked bar plot quantifying the proportional shift between *Cd274*^+^ (gene expression greater than 0) and *Cd274*^−^ MDSCs over time. (K) Schematic of spleen photoconversion in Kaede mice. (L to N) Representative FCM plots (L) and percentages (M and N) of Kaede red expression in PD-L1^+^ PMN-MDSCs in lung (*n* = 4 to 6). Values are mean ± SD. Comparisons were analyzed with Student *t* test; **P < 0.01.

In the lungs, total MDSCs and PMN-MDSCs accumulated continuously over 7 d (Fig. 1P and Q). PD-L1^+^ PMN-MDSCs constituted the majority (>90%) of PD-L1^+^ MDSCs in the lung and peaked at day 3 (Fig. 1R, S, U, and V). M-MDSCs, though fewer, also increased steadily through day 7 (Fig. 1R and T). Previously, we reported that small intestinal γδ T cells can migrate to the lungs within 1 d after CLP [13]. The opposing temporal patterns of PD-L1^+^ PMN-MDSCs in the spleen (early peak and decline) and lung (delayed, sustained rise) led us to hypothesize that these cells migrate from spleen to lungs during early sepsis. To test this, we used Kaede-transgenic (Kaede-tg) mice, in which localized UV irradiation photoconverted splenic cells from green to red fluorescence (Fig. 2A and B), enabling migration tracking.

To further characterize splenic-origin PD-L1^+^ PMN-MDSCs migrating to the lung, we performed single-cell RNA sequencing (scRNA-seq) on Kaede-red^+^ CD45^+^ cells sorted from lung tissue at 12 h, 1 d, 3 d, and 7 d post-CLP. Unsupervised clustering of the integrated dataset revealed diverse spleen-derived immune populations in the lung, including lymphocytes, monocytes, macrophages, and neutrophils (Fig. 2C and Fig. S1A). Subsetting and reclustering of the myeloid compartment identified clusters 0 and 1 as PMN-MDSC clusters on the basis of enrichment of established MDSC-associated transcriptional programs together with expression of granulocytic genes, including Csf3r and Cxcr2 (Fig. 2D and Fig. S1C). Because transcriptomic signatures alone do not establish suppressive function, this classification was interpreted together with independent functional assays performed on the corresponding flow-cytometrically defined splenic population. Further subclustering resolved 6 distinct MDSC subpopulations defined by top marker genes: *Ngp*^+^, *Junb*^+^, *Hmox1*^+^, *Il1b*^+^, *Il1rn*^+^, and *Cxcr2*^+^ MDSCs (Fig. 2E and F).

Notably, *Ngp*^+^ and *Il1b*^+^ MDSCs exhibited enhanced chemotaxis and migration signatures (Fig. 2G) and were more abundant at day 3 post-CLP (Fig. 2H), indicating greater migratory capacity at this time. Functional analysis revealed distinct properties: *Ngp*^+^ MDSCs showed elevated metabolic and cell cycle activity, suggesting a proliferative, immature state (Fig. S1D). This cluster was the primary source of *Cybb* (encoding NOX2), predicting ROS-mediated immunosuppression (Fig. S1E), and highly expressed *S100a8* and *S100a9*, indicating inflammatory origin and amplification (Fig. S1F). *Ngp*^+^ MDSCs were also the main producer of *Lipocalin-2* (*Lcn2*), suggesting immune regulation via iron metabolism. In contrast, *Il1b*^+^ MDSCs highly expressed *Il1b* and *Entpd1* (Fig. S1E), representing a more terminally differentiated immunosuppressive subpopulation.

This functional divergence was corroborated by pseudotime trajectory analysis, which positioned *Ngp*^+^ MDSCs at the start of the developmental path, with *Il1b*^+^ and *Il1rn*^+^ MDSCs at terminal branches (Fig. S1G). Cells from 12-h and 1-d post-CLP groups were enriched in initial segments, while those from 3- and 7-d groups localized to terminal segments (Fig. S1H). All subpopulations uniformly expressed high *Ly6g* levels (Fig. 2I), supporting their granulocytic lineage. Critically, the proportion of *Cd274*^+^ PMN-MDSCs peaked at day 3 (Fig. 2J), indicating the maximum pulmonary migration of PD-L1^+^ PMN-MDSCs. Flow cytometry validated that over 80% of PD-L1^+^ PMN-MDSCs in the lungs originated from the spleen by day 3 post-CLP, supporting early splenic-to-pulmonary migration (Fig. 2K to N).

**Fig. 3.**
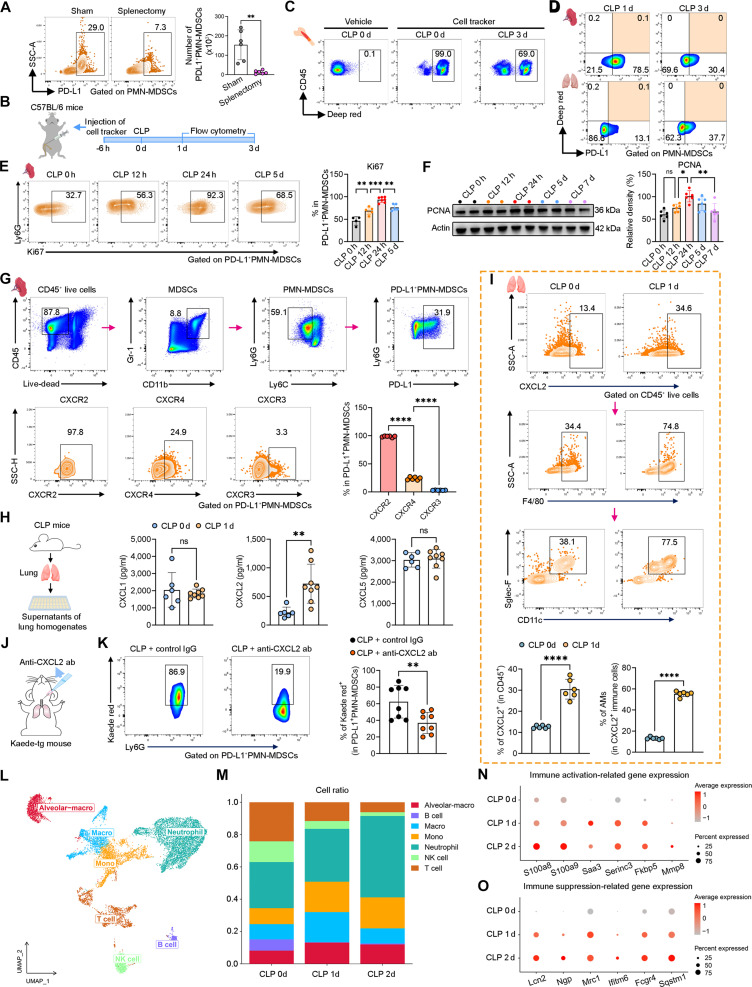
AM-derived CXCL2 recruits splenic CXCR2^+^ PD-L1^+^ PMN-MDSCs to lung. (A) Representative FCM plots and quantification of PD-L1^+^PMN-MDSCs in lungs (*n* = 6). (B) Design for labeling bone marrow cells. (C) Example FCM plots showing Cell-Tracker Deep Red labeling in bone marrow CD45^+^ cells. (D) Example FCM plots assessing labeled PD-L1^+^ PMN-MDSCs in lung and spleen. (E) Ki67 staining in splenic PD-L1^+^ PMN-MDSCs with quantification (*n* = 4 to 7). (F) PCNA protein abundance in sorted splenic PD-L1^+^ PMN-MDSCs from CLP mice (*n* = 6). (G) FCM gating and quantification of chemokine receptors on splenic PD-L1^+^ PMN-MDSCs (*n* = 6). (H) ELISA measurement of CXCL1, CXCL2, and CXCL5 (*n* = 6 to 8). (I) FCM analysis of CXCL2^+^ CD45^+^ cells and AM contribution to the CXCL2^+^ immune-cell fraction (*n* = 6). (J) Intratracheal anti-CXCL2 antibody scheme. (K) Example FCM plots and quantification of Kaede-red^+^ PD-L1^+^ PMN-MDSCs in lung (*n* = 8). (L) Integrated UMAP of lung immune cells from CLP mice. (M) Immune-cell frequencies. (N and O) Expression of activation-related (N) and suppression-related (O) genes in AMs. Values are mean ± SD. Panels (A), (H), (I), and (K) were analyzed by Student *t* test; panels (E) to (G) were analyzed by one-way ANOVA with Tukey’s post hoc test. *P < 0.05, **P < 0.01, ***P < 0.001, ****P < 0.0001. ns, not significant.

To conclusively determine the necessity of the spleen in this migratory circuit, we subjected mice to a splenectomy 14 d prior to CLP. Consistent with our Kaede tracing results, surgical removal of the spleen significantly reduced the accumulation of PD-L1^+^ PMN-MDSCs in the lungs at day 3 post-sepsis (Fig. 3A). Given the established role of the bone marrow as a major source of MDSCs [29–31], we asked whether splenic PD-L1^+^ PMN-MDSCs originate from the bone marrow. We tracked fluorescently labeled bone marrow cells at days 0, 1, and 3 post-CLP (Fig. 3B). Although robust bone marrow labeling was confirmed (Fig. 3C), virtually no PD-L1^+^ PMN-MDSCs derived from the bone marrow (~0%) were detected in the lung at either day 1 or day 3 post-CLP (Fig. 3D). This stands in stark contrast to our Kaede-tracking data, which demonstrated that approximately 80.5% of pulmonary PD-L1^+^ PMN-MDSCs are spleen-derived by day 3. While these distinct tracking methodologies, utilizing transgenic Kaede mice for splenic tracking versus exogenous dye labeling in wild-type (WT) C57BL/6 mice for bone marrow tracking, preclude simultaneous detection within a single flow cytometric assay, this comparative quantification unequivocally establishes the spleen, rather than the bone marrow, as the dominant extramedullary source for this early wave of pulmonary MDSCs. Furthermore, no bone marrow-derived cells were detected in the spleen, indicating that the early splenic increase in these cells does not result from bone marrow migration. Instead, we considered local proliferation as a potential mechanism. Flow-sorting of splenic PD-L1^+^ PMN-MDSCs revealed elevated expression of Ki67 and PCNA, peaking at day 1 post-CLP (Fig. 3E and F). This supports the notion that early expansion of this immunosuppressive population in the spleen arises from local proliferation in response to systemic inflammation.

### CXCL2–CXCR2 axis mediates migration of PD-L1^+^ PMN-MDSCs to the lung

We next sought to identify signals that guide splenic PD-L1^+^ PMN-MDSCs to the lung during early sepsis. Because chemokine pathways are central regulators of immune-cell trafficking [32], we screened chemokine receptors on splenic PD-L1^+^ PMN-MDSCs by flow cytometry. CXCR2 was the dominant receptor on this subset (Fig. 3G). In mice, CXCR2 responds mainly to CXCL1, CXCL2, and CXCL5 [33], and among these ligands, CXCL2 was significantly increased in lung homogenates at day 1 after CLP (Fig. 3H). AMs represented the main CXCL2-producing immune-cell population (Fig. 3I), consistent with their recognized role in pulmonary chemokine production [34]. Blocking CXCL2 by intratracheal antibody delivery sharply decreased recruitment of spleen-derived PD-L1^+^ PMN-MDSCs into the lung at day 3 (Fig. 3J and K), establishing CXCL2–CXCR2 signaling as a key migratory pathway.

We further characterized AMs during early sepsis using public scRNA-seq data, which revealed an increase in their proportion at day 1, followed by a slight decline by day 2 (Fig. 3L and M). Transcriptomic profiling indicated concurrent up-regulation of both immune activation and immunosuppressive gene programs (Fig. 3N and O), reflecting a dual functional phenotype.

Building on previous evidence of Wnt pathway activation in AMs during sepsis [13]. We stimulated MH-S cells with lipopolysaccharide (LPS) and confirmed Wnt activation via increased LEF1 expression, which was inhibited by the Wnt antagonist iCRT14 (Fig. S2A). Chromatin immunoprecipitation assay with sequencing (ChIP-seq) in MH-S cells identified LEF1 binding near the promoter of Kruppel-like factor 9 (KLF9) (Fig. S2B and C), a transcription factor associated with apoptosis regulation [35]. Given the reported association between PD-1 and immune exhaustion, we investigated whether KLF9 might modulate PD-1 expression in AMs. Both KLF9 and PD-1 expression levels were up-regulated following LPS stimulation and were markedly reduced upon Wnt pathway inhibition with iCRT14 (Fig. S2D). KLF9 expression correlated positively with PD-1 expression (Fig. S2E), and co-immunoprecipitation (co-IP) demonstrated a physical association between KLF9 and PD-1 proteins (Fig. S2F). Lentiviral knockdown of KLF9 reduced PD-1 in AMs (Fig. S2G and H), supporting KLF9 as a mediator connecting Wnt signaling to PD-1 induction during early sepsis. To distinguish direct transcriptional regulation from protein–protein interactions, we conducted dual-luciferase reporter assays. LEF1 overexpression robustly transactivated the WT *Klf9* promoter, whereas mutating the LEF1-binding consensus completely abrogated this response (Fig. S2I). Similarly, KLF9 specifically transactivated the WT *Pdcd1* promoter but failed to activate the corresponding binding-site mutant (Fig. S2J). Coupled with our co-IP data, these findings establish a definitive Wnt/LEF1–KLF9–PD-1 transcriptional axis underlying early AM immunoparalysis.

### PD-L1^+^ PMN-MDSC recruitment impairs pulmonary immunity via contact-dependent macrophage reprogramming

To assess the functional impact of splenic PD-L1^+^ PMN-MDSC lung recruitment, we isolated AMs and PMN-MDSCs at the indicated time points after CLP (Fig. S3A). To functionally validate the classification of the sorted population as PMN-MDSCs, we performed a classical T-cell suppression assay. Fluorescence-activated cell sorting (FACS)-sorted splenic PMN-MDSCs from mice 24 h after CLP were cocultured with anti-CD3/CD28-stimulated, carboxyfluorescein succinimidyl ester (CFSE)-labeled responder cells for 3 d. The addition of PMN-MDSCs significantly inhibited T-cell proliferation compared with stimulated responder cells cultured without PMN-MDSCs (Fig. S3B).

To further characterize the suppressive phenotype of these PMN-MDSCs beyond surface phenotyping, we assessed intracellular immunosuppressive enzyme expression and reactive molecule production. Separately, analysis of the corresponding Live CD45^+^ CD11b^+^ Gr-1^+^ Ly6G^hi^ Ly6C^lo^ splenic compartment at day 3 after CLP demonstrated significantly higher Arg-1 expression than phenotype-matched splenic cells from sham mice, which served as the same-tissue steady-state neutrophil control (Fig. S3C). The CLP-derived population also exhibited markedly elevated basal ROS and NO production relative to the sham control population (Fig. S3D and E). Along with sepsis progression, both PD-1 on AMs and PD-L1 on PMN-MDSCs increased (Fig. S3F). In direct coculture using the murine AM MH-S cell line, LPS-stimulated MH-S cells exposed to PD-L1^+^ PMN-MDSCs showed elevated PD-1 and KLF9, increased interleukin-10 (IL-10) secretion, and enhanced M2 polarization (Fig. S3G to O), with these immunosuppressive markers strongly correlating (Fig. S3P), suggesting MDSC-induced AM reprogramming.

In a sepsis model followed by secondary *Pseudomonas aeruginosa* infection, adoptive transfer of PD-L1^+^ PMN-MDSCs into the lungs significantly reduced survival (Fig. S3Q). Notably, while the endogenous peak of pulmonary PD-L1^+^ PMN-MDSCs reaches approximately 1 × 10^5^ cells, an intratracheal instillation dose of 2 × 10^5^ cells was specifically employed. This approach intentionally offsets the expected cellular attrition and mucociliary clearance inherent to airway delivery, ensuring that the final functional engraftment within the alveolar space accurately mimics a severe pathophysiological burden without representing an artifactual extreme. Recipient mice showed impaired pulmonary immunity, with reduced alveolar immune-cell infiltration, fewer neutrophils, and lower bronchoalveolar lavage fluid (BALF) concentrations of IL-6, IL-17A, and tumor necrosis factor-α (TNF-α) (Fig. S3R to T), indicating that PD-L1^+^ PMN-MDSCs foster an immunosuppressive microenvironment that compromises host defense.

To determine the mechanism of MDSC–AM crosstalk, we used a Transwell system to separate direct contact from soluble factor-mediated effects. LPS-stimulated MH-S cells cocultured with physically separated PD-L1^+^ PMN-MDSCs did not up-regulate PD-1 or IL-10, or alter polarization (Fig. S4A to F). In contrast, direct contact suppressed MH-S phagocytosis and pro-inflammatory cytokine production (Fig. S4G and H). To further ensure the physiological relevance of these findings, we replicated these assays using primary murine AMs isolated from BALF, with high purity confirmed by flow cytometry (Fig. S4I). In a direct coculture setting, exposure to PD-L1^+^ PMN-MDSCs significantly increased the expression of PD-1, IL-10, and C1q in LPS-stimulated primary AMs compared to LPS stimulation alone (Fig. S4J and L). Strikingly, physically separating the primary cells in a Transwell system completely abrogated these up-regulations, yielding no significant differences between the groups (Fig. S4K and M). Together, these results definitively indicate that PD-L1^+^ PMN-MDSC-mediated immunosuppression is strictly contact-dependent.

**Fig. 4.**
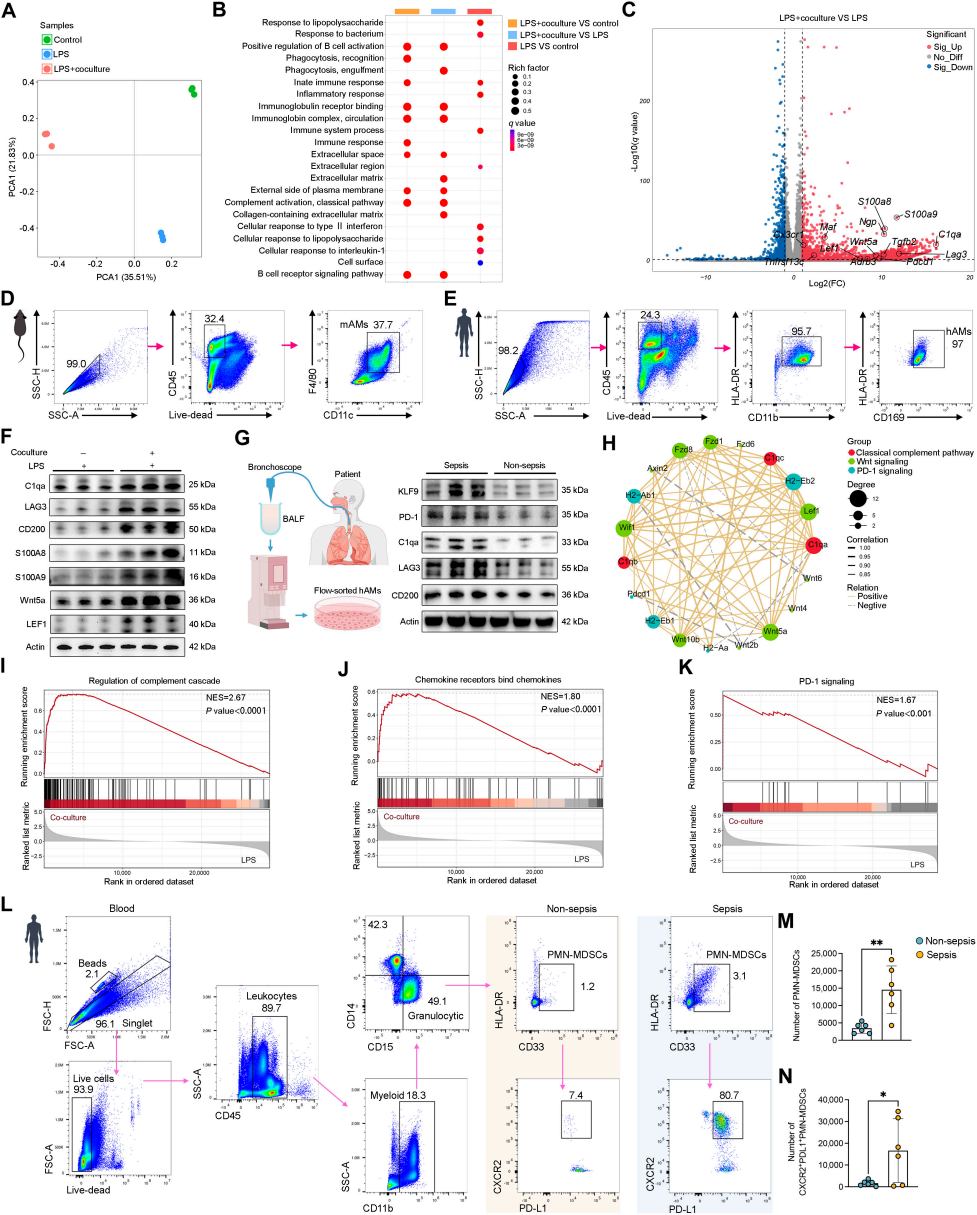
LPS-stimulated AMs cocultured with PD-L1^+^ PMN-MDSCs exhibit immunosuppressive and exhausted profiles. (A) Principal component analysis (*n* = 3). (B) GO enrichment analysis. (C) Volcano plot of differentially expressed genes. (D) Flow cytometric sorting strategy for murine alveolar macrophages. (E) Flow cytometric sorting strategy for alveolar macrophages from bronchoalveolar lavage fluid (BALF) of clinical patients. (F) Protein levels of C1q, LAG3, CD200, S100A8, S100A9, Wnt5a, and LEF1 in LPS-stimulated AMs cocultured or not with PD-L1^+^ PMN-MDSCs. (G) Protein levels of KLF9, PD-1, C1q, LAG3, and CD200 in flow-sorted AMs from BALF of septic and nonseptic patients. (H) Correlation network of PD-1, Wnt, and classical complement pathways. (I to K) GSEA of complement regulation (I), chemokine receptor binding (J), and PD-1 signaling (K). (L) Flow cytometric gating strategy for identifying PMN-MDSCs (defined as CD45^+^ CD11b^+^ CD15^+^ CD14^−^ CD33^+^ HLA-DR^l^°^w^) and the CXCR2^+^ PD-L1^+^ subpopulation in the peripheral blood of clinical patients. (M and N) Absolute number of total PMN-MDSCs (M) and CXCR2^+^ PD-L1^+^ PMN-MDSCs (N) in peripheral blood from septic and nonseptic patients (*n* = 6). Values are mean ± SD. Panels (M) and (N) were analyzed by Student *t* test. *P < 0.05, **P < 0.01.

We then used RNA-seq to define the transcriptional changes associated with AM immunosuppression. AMs were compared across 3 conditions: untreated controls, LPS stimulation alone, and LPS stimulation with PD-L1^+^ PMN-MDSC coculture. Principal component analysis (PCA) tightly separated the 3 groups (untreated controls, LPS alone, and LPS + coculture; *n* = 3 independent biological replicates per group) along distinct principal components (Fig. 4A). The robust intergroup separation and minimal within-group variation underscore the high reproducibility and reliability of the transcriptomic shifts induced by direct MDSC coculture. While LPS-stimulated AMs displayed enriched gene signatures related to innate immune activation and inflammation, cocultured AMs exhibited attenuated pro-inflammatory signaling and robust activation of the classical complement pathway (Fig. 4B). Among the genes significantly up-regulated in the coculture condition, several were of particularly immunological interest. *Wnt5a* and *Lef1*, 2 key components of the Wnt signaling pathway, were prominently increased. *S100a8* and *S100a9*, which form heterodimers with roles in infection, inflammation, and immune modulation [36], were also notably elevated. Of special relevance, *C1qa*, a major subunit of the C1q hexamer that initiates the classical complement cascade [37], was significantly up-regulated. Recent studies have implicated C1q in promoting immunosuppressive phenotypes in macrophages [38]. In parallel, we observed increased expression of several well-established immune checkpoint and exhaustion-related molecules, including *Pdcd1* (encoding PD-1), *Lag3*, and *Cd200* (Fig. 4C), findings validated at the protein level (Fig. 4D and F). Similar increases in KLF9, PD-1, C1qa, LAG3, and CD200 were detected in AMs isolated from septic patients compared with nonseptic controls (Fig. 4E and G), supporting clinical relevance. Pathway and network analyses indicated interconnected complement, Wnt, and PD-1 signaling modules (Fig. 4H), and Gene Set Enrichment Analysis (GSEA) further supported enrichment of complement regulation, chemokine–receptor interactions, and PD-1 signaling in cocultured AMs (Fig. 4I to K).

Beyond the local pulmonary environment, we sought to validate the systemic mobilization of these suppressive cells in humans. Flow cytometric analysis of peripheral blood demonstrated that septic patients exhibited significantly increased number of total PMN-MDSCs and the specific CXCR2^+^ PD-L1^+^ PMN-MDSC subset relative to nonseptic controls (Fig. 4L to N). This systemic expansion of functional MDSCs, coupled with the exhausted phenotype of lung-resident AMs, provides robust clinical evidence supporting the existence of this interorgan immunosuppressive pathway.

To determine whether PD-1 defines a functionally distinct AM subset, we sorted cocultured AMs into PD-1^high^ and PD-1^low^ populations. PD-1^high^ AMs exhibited elevated LAG3, C1q, and CD200, confirming a distinct immunosuppressive phenotype (Fig. S5A to I). Total AM numbers declined after coculture (Fig. S5M), and a similar PD-1^high^ subset expressing exhaustion markers was identified in AMs from day 3 post-CLP mice (Fig. S6A to G). The proportion of AMs among lung immune cells increased transiently on day 1 but dropped by day 3 (Fig. S6H), suggesting that PD-L1^+^ PMN-MDSCs drive both functional reprogramming and attrition of AMs, facilitating an immunosuppressive niche in sepsis.

### PD-1 deficiency in AMs attenuates immunosuppression during sepsis

**Fig. 5.**
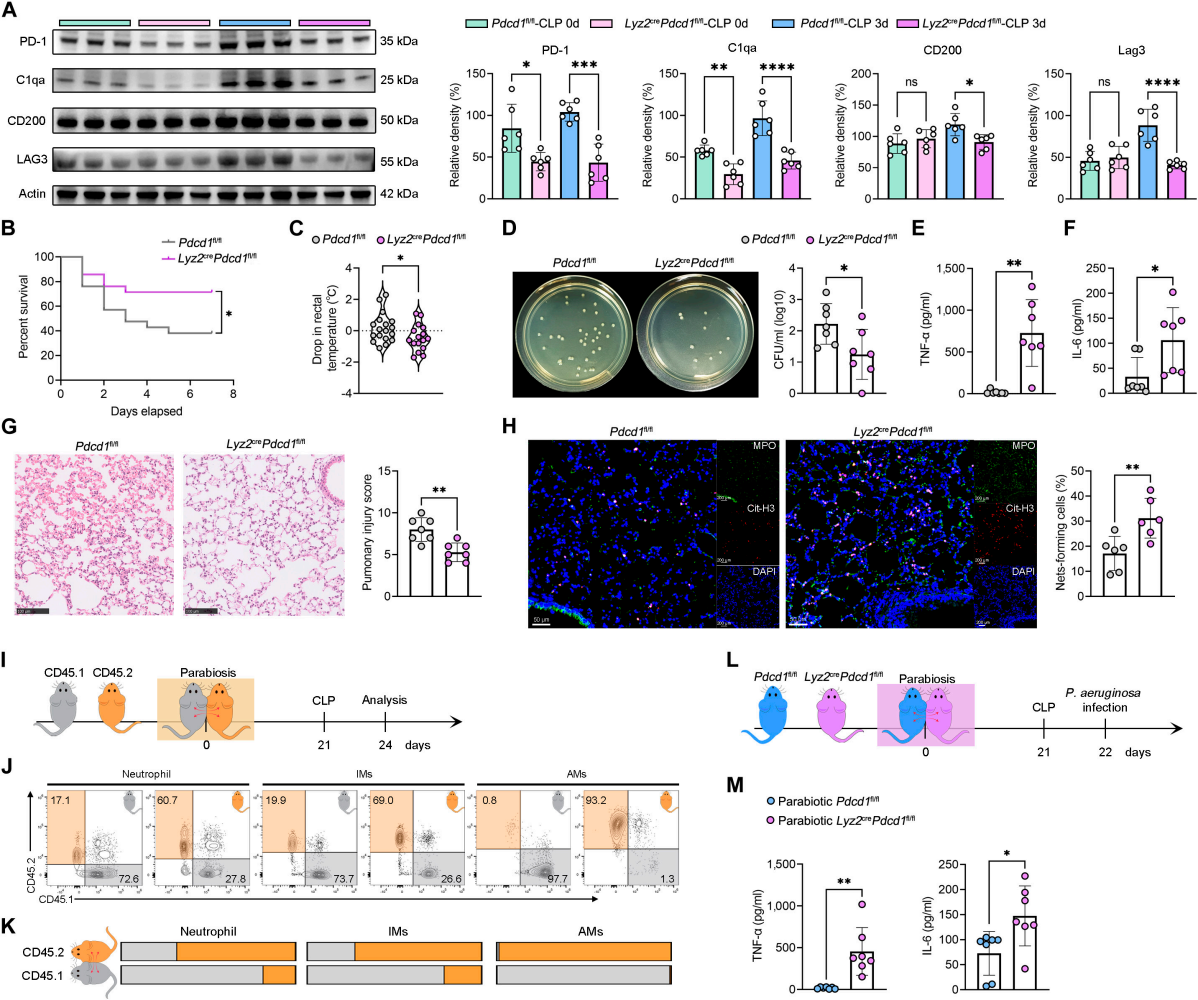
Myeloid PD-1 deficiency improves outcomes after polymicrobial sepsis and *Pseudomonas aeruginosa* infection. (A) Protein levels of PD-1, C1qa, CD200, and LAG3 in flow-sorted AMs from *Pdcd1*^flox/flox^ and *Lyz2*^Cre^ *Pdcd1*^flox/flox^ mice post-CLP (*n* = 6). (B) Kaplan–Meier survival analysis following *P. aeruginosa* infection (*n* = 21). (C to F) Core body temperature (*n* = 16 to 18) (C), bacterial lung burden (*n* = 7) (D), and BALF levels of TNF-α (*n* = 7) (E) and IL-6 (*n* = 7) (F), in septic mice 1 d after *P. aeruginosa* infection. (G) Lung H&E images and histological injury scoring (*n* = 7). (H) Immunofluorescence images and semiquantitative analysis of NET formation using Cit-H3 and MPO staining (*n* = 6). Scale bar, 50 μm. (I) Parabiosis and CLP workflow. (J and K) Example FCM plots (J) and proportions of partner-derived neutrophils, IMs, and AMs in lung (*n* = 5) (K). (L) Schematics of parabiosis of *Pdcd1*^flox/flox^ and *Lyz2*^Cre^ *Pdcd1*^flox/flox^ mice. (M) BALF TNF-α and IL-6 in septic parabionts after *P. aeruginosa* infection (*n* = 7). Values are mean ± SD. Panel (A) was analyzed by one-way ANOVA with Tukey’s post hoc test; panel (B) was analyzed by log-rank (Mantel–Cox) test; panels (C) to (H) and (M) were analyzed by Student *t* test. *P < 0.05, **P < 0.01, ***P < 0.001, ****P < 0.0001. ns, not significant.

Given the observed up-regulation of PD-1 in AMs during early sepsis, we examined its functional role by generating myeloid-specific PD-1-deficient mice through crossing *Lyz2*^cre^ mice with *Pdcd1*^flox/flox^ mice. At 3 d post-CLP, *Lyz2*^Cre^ *Pdcd1*^flox/flox^ mice exhibited significantly reduced expression of the immunosuppressive markers LAG3, C1q, and CD200 in AMs compared to control *Pdcd1*^flox/flox^ mice (Fig. 5A). Functionally, these mice displayed enhanced resistance to *P. aeruginosa* secondary pulmonary infection, as evidenced by reduced mortality (Fig. 5B), more rapid recovery of body temperature (Fig. 5C), and superior bacterial clearance from the lungs (Fig. 5D). Consistent with these improvements, *Lyz*^Cre^ *Pdcd1*^flox/flox^ mice mounted stronger pulmonary immune responses, including elevated BALF concentrations of pro-inflammatory cytokines TNF-α and IL-6 (Fig. 5E and F), reduced histopathological lung injury (Fig. 5G), and increased neutrophil infiltration into the lung (Fig. 5H).

Lyz2-Cre activities span multiple myeloid subsets, including AMs, interstitial macrophages (IMs), monocytes, and neutrophils [39]. To distinguish whether the loss of PD-1 in AMs specifically contributes to pulmonary immune reprogramming, we performed parabiosis experiments between *Pdcd1*^flox/flox^ and *Lyz2*^Cre^ *Pdcd1*^flox/flox^ mice (Fig. 5I). First, to confirm successful vascular integration and immune cell exchange, we joined congenic CD45.1^+^ and CD45.2^+^ mice. Twenty-one days post-parabiosis, both partners underwent CLP, and after 3 d, lung tissues were analyzed. Notably, substantial proportions of neutrophils and IMs were of parabiotic origin (Fig. 5J and K), whereas AMs remained largely host-derived, with <2% originating from the partner, even during sepsis (Fig. 5J and K). These findings suggest that AMs are maintained through local self-renewal and are minimally replenished by circulating monocytes in the early septic phase.

We next subjected *Pdcd1*^flox/flox^ and *Lyz2*^Cre^ *Pdcd1*^flox/flox^ parabiotic pairs to the 2-hit model of CLP followed by *P. aeruginosa* infection (Fig. 5L). At 1 d post-secondary infection, *Lyz2*^Cre^ *Pdcd1*^flox/flox^ mice exhibited significantly elevated BALF levels of TNF-α and IL-6 relative to their *Pdcd1*^flox/flox^ partners (Fig. 5M), indicating more robust pulmonary immune activation. Together, these results support the conclusion that PD-1 deficiency in AMs, rather than in circulating monocytes or neutrophils, drives improved host defense, likely through a cell-autonomous mechanism that restores AM immune competence during sepsis.

To further assess the therapeutic potential of targeting PD-1, we performed in vitro cocultures of AMs and PD-L1^+^ PMN-MDSCs under LPS stimulation. Blocking PD-1 with anti-PD-1 antibodies significantly restored AM proliferative capacity (Fig. S7A). This was accompanied by a marked reduction in immunosuppressive and exhaustion markers (C1q, LAG3, CD200, IL-10, and transforming growth factor-β [TGF-β]), and a concurrent increase in pro-inflammatory cytokines TNF-α and IL-6 (Fig. S7B). Given that splenic PD-L1^+^ PMN-MDSCs migrate into the lungs and drive PD-1 up-regulation in AMs during early sepsis (day 3), we administered airway-delivered anti-PD-1 antibodies at the time of sepsis induction (Fig. S7C). This intervention not only restored AM numbers at day 3 post-sepsis but also reduced C1q, LAG3, and CD200 expression levels (Fig. S7D to H). Furthermore, anti-PD-1 treatment significantly improved survival following secondary *P. aeruginosa* infection (Fig. S7I), alleviated lung injury (Fig. S7J), and enhanced pulmonary immune responses, as evidenced by elevated TNF-α, IL-6 and IFN-γ in BALF (Fig. S7K and L).

Expanding on these findings, RNA-seq analyses revealed that AM coculture with PD-L1^+^ PMN-MDSCs robustly activated the complement pathway and elevated C1q expression. To evaluate whether targeting C1q during early sepsis could improve outcomes, we administered intratracheal anti-C1q antibodies immediately post-sepsis induction (Fig. S8A). This intervention significantly improved survival following secondary *P. aeruginosa* challenge (Fig. S8B); increased BALF levels of IL-6, IFN-γ, and TNF-α (Fig. S8C); and decreased pulmonary expression of immunosuppressive markers including LAG3, CD200, IL-10, and TGF-β (Fig. S8D). Notably, while C1q blockade successfully reversed downstream immunoparalysis, PD-1 expression remained unchanged (Fig. S8D). Given our earlier findings that PD-1 deficiency or pharmacological PD-1 blockade dramatically suppressed C1qa expression, this unidirectional intervention response definitively places C1q downstream of the PD-1 signaling axis in the immunosuppressive cascade.

Mechanistic insights from RNA-seq analyses further indicated crosstalk between the complement and Wnt signaling pathways. Co-IP assays revealed a physical interaction between the Wnt transcription factor LEF1 and C1qa (Fig. S8E). Pharmacological inhibition of Wnt signaling using iCRT14 significantly reduced C1qa expression (Fig. S8F), indicating that LEF1 promotes complement activation in sepsis via transcriptional regulation of C1qa. These findings delineate a sequential regulatory axis in which early Wnt activation drives LEF1-mediated C1qa expression, which in turn amplifies PD-1-dependent immunosuppression in AMs during sepsis pathogenesis.

### Epigenetic and transcriptional reprogramming drives PD-L1^+^ PMN-MDSC expansion and immunosuppression in early sepsis

**Fig. 6.**
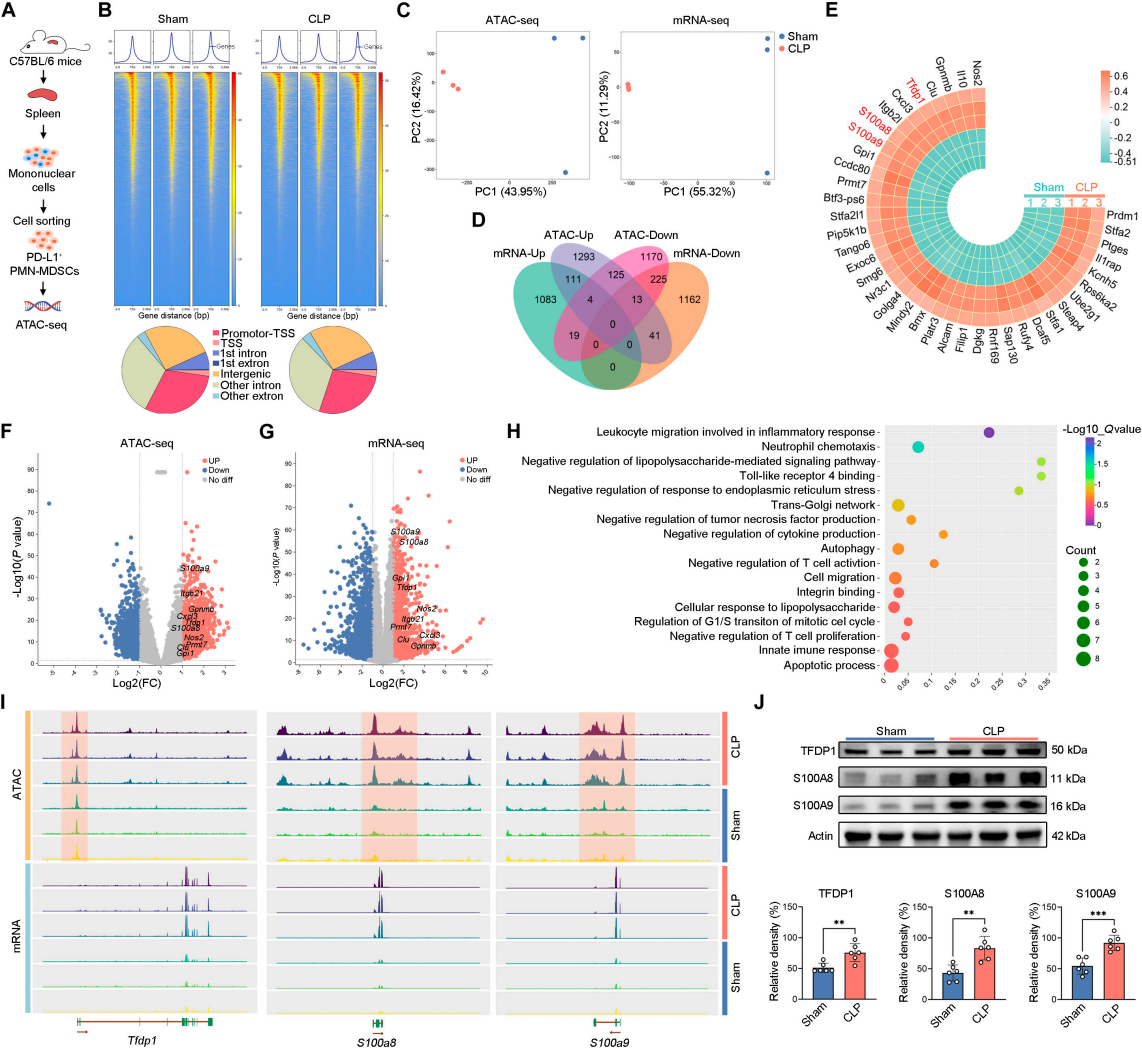
ATAC-seq and RNA-seq profiling of splenic PD-L1^+^ PMN-MDSCs. (A) Tissue processing and sequencing workflow. (B) Top panel: TSS enrichment and average read depth; bottom panel: genomic distribution of differentially accessible ATAC-seq peaks. (C) PCA of ATAC-seq and mRNA-seq datasets. (D) Venn diagram showing genes with coordinated changes in chromatin accessibility and mRNA expression. (E) Heatmap of genes up-regulated at both chromatin and transcript levels. (F and G) Volcano plots for ATAC-seq (F) and mRNA-seq (G). (H) KEGG enrichment for overlapping differentially expressed genes. (I) Chromatin accessibility and mRNA abundance of *Tfdp1*, *S100a8*, and *S100a9*. (J) TFDP1, S100A8, and S100A9 protein levels in splenic PD-L1^+^ PMN-MDSCs (*n* = 6). Values are mean ± SD. Panel (J) was analyzed by Student *t* test. **P < 0.01, ***P < 0.001.

To investigate how splenic PD-L1^+^ PMN-MDSCs proliferate and acquire suppressive activity at day 1 after sepsis, we sorted these cells from CLP and sham spleens (Fig. 6A). We then profiled transcriptional regulation at the chromatin level using paired Assay for Transposase-Accessible Chromatin with high-throughput sequencing (ATAC-seq) and RNA-seq. ATAC-seq showed strong enrichment of accessible regions around transcription start sites (TSSs), with comparable sequencing depth across CLP and control samples (Fig. 6B). More than 25% of ATAC-seq peaks mapped to promoter-proximal regions within ±3 kb of TSSs (Fig. 6B). PCA of both ATAC-seq and RNA-seq datasets clearly separated septic from control samples, indicating broad epigenetic and transcriptional remodeling after CLP (Fig. 6C).

To directly associate chromatin accessibility with transcriptional changes, we performed an integrative Venn analysis. This identified 111 genes with concurrent increases in chromatin accessibility and mRNA expression, and 225 genes showing concordant down-regulation (Fig. 6D). Among the up-regulated genes, we noted several with established roles in cell adhesion and migration, including *Ccdc80*, *Itgb2l*, *Cxcl3*, *S100a8*, and *S100a9* (Fig. 6E to G). Notably, *S100a8* and *S100a9* have been previously implicated in the up-regulation of PD-L1. Additionally, *Tfdp1*, a transcription factor essential for the G1-to-S phase transition during the cell cycle [40], emerged as a key gene with increased chromatin accessibility and transcription in the CLP group, suggesting a potential role in enhancing PMN-MDSC proliferation and accumulation in the lungs.

Together, these data delineate a bifurcated regulatory program within the splenic niche: TFDP1 strictly drives the cellular proliferation and local expansion of PMN-MDSCs, whereas the concurrent up-regulation of S100A8/S100A9 strengthens their suppressive phenotype, specifically by promoting PD-L1 expression. Gene ontology (GO) analysis of accessible regions revealed enrichment of leukocyte migration and negative regulation of LPS-mediated signaling (Fig. 6H), consistent with their immunomodulatory function. Both chromatin accessibility and expression levels of *Tfdp1*, *S100a8*, and *S100a9* were significantly higher in the CLP group relative to controls (Fig. 6I), and these up-regulations were corroborated at the protein level in splenic PD-L1^+^ PMN-MDSCs at day 1 post-sepsis (Fig. 6J).

**Fig. 7.**
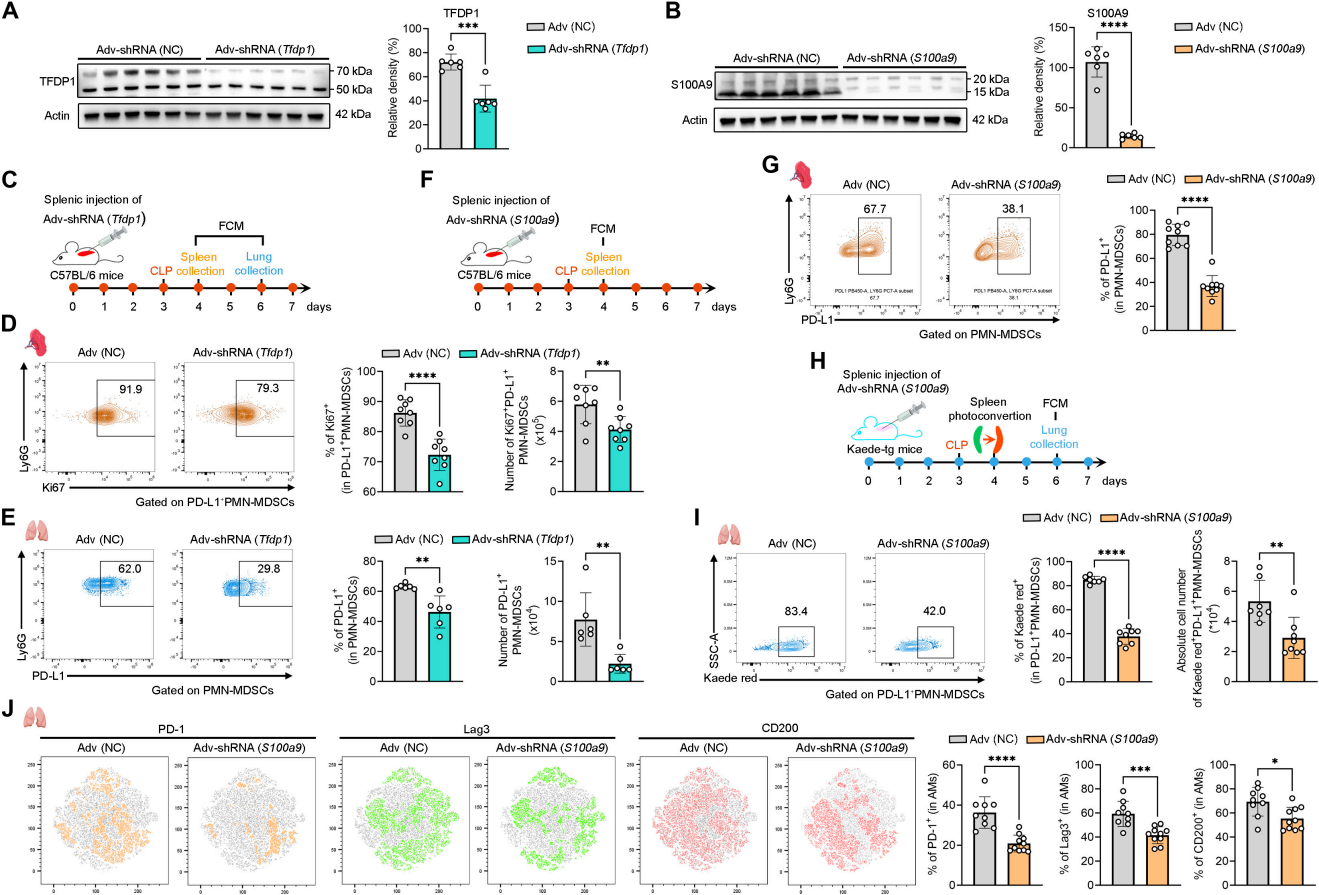
TFDP1 and S100A9 promote expansion and immunosuppression of splenic PD-L1^+^ PMN-MDSCs in early sepsis. (A and B) Western blot and quantification of TFDP1 (A) (*n* = 6) and S100A9 (B) (*n* = 6) in spleens. (C) Schematic of CLP modeling, Adv-shRNA (Tfdp1) treatment, and tissue collection in WT mice. (D) Example FCM plots and percentage of Ki67 in splenic PD-L1^+^ PMN-MDSCs (*n* = 8). (E) Example FCM plots and percentage of PD-L1 in pulmonary PMN-MDSCs (*n* = 6). (F) Schematic of CLP modeling, Adv-shRNA (*S100a9*) treatment, and tissue collection in WT mice. (G) Example FCM plots and percentage of PD-L1 in splenic PMN-MDSCs (*n* = 9). (H) Schematic of CLP modeling, Adv-shRNA (*S100a9*) treatment, and tissue collection in Kaede-tg mice. (I) Example FCM plots and percentage of Kaede red^+^ in pulmonary PD-L1^+^ PMN-MDSCs (*n* = 7 to 8). (J) FCM analysis using t-SNE algorithm showing expressions of PD-1, Lag3, and CD200 in pulmonary AMs (*n* = 9 to 10). Values are shown as mean ± SD; statistical testing was determined using Student *t* test. *P < 0.05, **P < 0.01, ***P < 0.001, ****P < 0.0001.

To move beyond correlative observations and establish the functional necessity of this upstream axis within the splenic niche, we performed targeted in vivo interventions using spleen-directed shRNA adenoviruses (Fig. 7A and B). Crucially, the intra-splenic knockdown of *Tfdp1* significantly reduced Ki67 positivity and dramatically curtailed the local expansion of splenic PD-L1^+^ PMN-MDSCs at day 1 post-sepsis (Fig. 7C to E). Similarly, targeted knockdown of *S100a9* suppressed the proportional accumulation of this subset in the septic spleen (Fig. 7F and G). Utilizing the Kaede-tg tracking system, we further demonstrated that suppressing *S100a9* in the spleen fundamentally impaired the emigration process, significantly reducing the absolute number of spleen-derived PD-L1^+^ PMN-MDSCs that successfully reached the pulmonary compartment (Fig. 7H and I). Consequently, resident AMs isolated from these knockdown mice exhibited significantly attenuated immunoparalysis, marked by lower expression levels of PD-1, LAG3, and CD200 (Fig. 7J). Together, these functional data seamlessly integrate our molecular findings, establishing the TFDP1/S100A9 axis as the upstream functional driver that licenses the expansion and subsequent lung-directed emigration of immunosuppressive PMN-MDSCs during early sepsis.

## Discussion

Sepsis-associated immunosuppression is often described as a transition from early hyperinflammation to later immune paralysis, but the spatial mechanisms that initiate this transition are not well understood. Our study identifies a spatiotemporally organized pathway in which splenic PD-L1^+^ PMN-MDSCs move to the lung through CXCL2/CXCR2 signaling and impair AM function through PD-L1/PD-1 contact-dependent interactions. Previous studies have associated MDSCs with sepsis immunosuppression; our data extend this framework by showing that, in early sepsis, the spleen rather than the bone marrow is the principal site of PD-L1^+^ PMN-MDSC expansion and that recruited cells directly reprogram AMs. These findings refine current models of sepsis immunopathology by placing interorgan myeloid communication upstream of lung-specific immune failure.

The spleen’s role as a hub for MDSC expansion contrasts with canonical models emphasizing bone marrow-derived myelopoiesis. While earlier work established that splenic CD11b^+^ Ly6C^high^ monocytes expand during later stage of sepsis [41], our data reveal that splenic PMN-MDSCs proliferate locally in early sepsis via TFDP1-driven mechanisms, independent of bone marrow input. This aligns with a previous study identifying spleen as a site for storage and rapid deployment of myeloid cells in regulation of inflammation [42]. The spleen’s unique microenvironment, which is rich in IL-3 and IL-6 after sepsis, likely supports TFDP1-mediated proliferation [43], a transcription factor previously linked to cell cycle progression in cancer [44]. Importantly, our findings suggest a precise division of labor during this splenic emergency myelopoiesis: while TFDP1 acts as an upstream license primarily for cellular expansion, the parallel S100A8/S100A9 axis is responsible for inducing the downstream suppressive phenotype and PD-L1 surface expression. Notably, our ATAC-seq data showing sepsis-induced chromatin remodeling at *Tfdp1* loci suggest epigenetic priming of splenic PMN-MDSCs for rapid expansion, a mechanism not yet reported in acute infection. This splenic dominance raises questions about organ-specific myeloid education. Unlike bone marrow MDSCs, which require prolonged exposure to cytokines like GM-CSF for immunosuppressive maturation [45], splenic PMN-MDSCs acquire PD-L1 expression within hours of sepsis onset. This accelerated phenotype may reflect the spleen’s role as a “first responder” to bloodborne pathogens, where immediate immunosuppressive signals prevent collateral tissue damage. However, this adaptation becomes maladaptive when MDSCs migrate to distal organs like the lungs, crippling antimicrobial defenses.

The spleen-to-lung migratory circuit underscores the importance of interorgan crosstalk in sepsis. The spleen, traditionally viewed as a site of lymphocyte activation and erythrocyte clearance, emerges as a coordinator of distal immune paralysis. This aligns with clinical observations that splenectomized sepsis patients exhibit altered immune trajectories [46], though the mechanistic basis was unclear. Our data suggest that splenic MDSCs act as “mobile immunosuppressive units”, deploying to organs like the lungs to enforce systemic tolerance. Crucially, this spleen–lung cellular conduit appears to be a fundamental mechanism of systemic immune orchestration rather than an isolated phenomenon in bacterial sepsis. A recent single-cell spatiotemporal mapping study identified a parallel “spleen-to-lung neutrophil axis” during SARS-CoV-2 infection [47]. In that viral context, the spleen functions as a key extramedullary hub that deploys neutrophils to replenish pulmonary pools and bolster innate antiviral defense. While their findings highlight the highly protective nature of interorgan myeloid trafficking during acute viral challenge, our study reveals its maladaptive subversion in sepsis through the targeted deployment of PD-L1^+^ PMN-MDSCs that cripple local antimicrobial defenses and establish immune paralysis. This mirrors findings in brain injury, where enhancing the activation of peripheral PMN-MDSC suppresses the neuroinflammation [48], but here, the interaction is uniquely dependent on PD-1/PD-L1 checkpoint signaling. Notably, the kinetics of MDSC migration, peaking at day 3 post-sepsis, coincides with the clinical transition from hyperinflammation to immunoparalysis. This temporal precision suggests that cross-organ migration is not a passive leakage of cells but an active process synchronized with disease progression. The CXCL2 gradient established by AMs likely serves as a “homing beacon”, akin to CXCL12-mediated hematopoietic stem cell trafficking [49]. However, in sepsis, this chemotactic signal is subverted to recruit immunosuppressive rather than protective cells.

CXCL2–CXCR2 signaling, which is classically associated with neutrophil recruitment in acute inflammation [50,51], is repurposed in sepsis to orchestrate the trafficking of immunosuppressive MDSCs. The dominance of CXCR2 on splenic PMN-MDSCs and their directional migration toward AM-derived CXCL2 redefine our understanding of chemokine biology in sepsis. While CXCL1 and CXCL2 are generally considered redundant neutrophil chemoattractants [52], our data suggest that in sepsis, AMs selectively up-regulate CXCL2 to establish a chemotactic gradient that preferentially attracts CXCR2^+^ PD-L1^+^ PMN-MDSCs. The nonredundant role of CXCR2 is further underscored by its enrichment over other chemokine receptors on these suppressive cells, positioning it as a central mediator of MDSC migration. By contrast, CXCR4, a chemokine receptor known for regulating hematopoietic stem cell trafficking [53], does not appear to participate in this immunosuppressive routing, reinforcing the specificity of the CXCL2–CXCR2 axis. From a translational perspective, it is noteworthy that while murine PD-L1^+^ PMN-MDSCs respond robustly to CXCL2–CXCR2 signaling, human PMN-MDSCs may rely more heavily on CXCL8 (IL-8) [54], a functional homolog of CXCL2 with higher affinity for human CXCR2. This species-specific ligand preference highlights the need for further investigation into CXCR2-mediated MDSC trafficking in human sepsis, which could inform the design of targeted chemokine receptor antagonists as potential immunomodulatory therapies.

Mechanistically, our data reveal a highly interconnected intracellular cascade linking the Wnt, PD-1, and classical complement pathways during AM immunoparalysis. Upon interaction with recruited MDSCs, AMs undergo profound epigenetic and transcriptional shifts. Initial inflammatory and contact-dependent stress activates Wnt signaling, leading to the accumulation of LEF1. Crucially, LEF1 promotes immune exhaustion through 2 interconnected avenues. First, LEF1 acts upstream to drive the transcription of KLF9, which we demonstrate physically interacts with and stabilizes PD-1. Second, our co-IP assays confirmed a physical interaction between LEF1 and C1qa, and pharmacological inhibition of Wnt signaling successfully reversed C1qa up-regulation. Although whether LEF1 regulates C1qa via direct transcriptional induction or posttranslational stabilization requires further investigation, this synchronized LEF1-mediated amplification of both C1q and PD-1 creates a feed-forward loop of immune exhaustion, definitively linking early innate Wnt activation to downstream checkpoint-induced immunoparalysis.

Clinically, our results provide a mechanistic explanation for why patients can remain vulnerable to secondary bacterial pneumonia even after the initial septic inflammation improves. The spleen–lung axis shows how extrapulmonary myeloid reprogramming can directly undermine AM-mediated host defense, a central barrier against sepsis-associated pneumonia and respiratory failure. The detection of PD-1^high^, complement-enriched AM features in human sepsis samples further supports the translational relevance of this pathway. These findings also complement recent research studies emphasizing macrophage reprogramming in sepsis immunotherapy and epithelial stress pathways in LPS-induced acute lung injury [55,56]. Rather than broadly stimulating immunity, lung-directed modulation of PD-1 or downstream complement signaling may provide a spatially targeted strategy to restore pulmonary defense while minimizing systemic immune activation. Such an approach is particularly relevant in intensive care, where preventing ventilator-associated and secondary bacterial pneumonia remains a major unmet need.

Translating these findings into clinical practice requires navigating substantial historical challenges. Systemic anti-PD-1/PD-L1 therapies have not yet achieved clinical success in human sepsis trials outside of oncology, likely due to patient heterogeneity, suboptimal timing of administration, or the pleiotropic risks of unconstrained hyperinflammation. Importantly, our study does not advocate for immediate systemic immune checkpoint blockade. Instead, our identification of a spatially restricted spleen–lung axis suggests that future therapeutic strategies might benefit from anatomic targeting, for example, localized aerosolized delivery of checkpoint inhibitors to the lung compartment, to disarm pulmonary immunosuppression while preserving systemic immune equilibrium. Thus, our current work is fundamentally mechanistic, providing a robust foundation for spatially defined interventions rather than broad systemic blockade.

PMN-MDSCs and activated neutrophils share granulocytic-lineage features and may overlap across developmental, transcriptional, and morphological states [57]. Accordingly, no single marker or morphological criterion used in this study should be interpreted as universally establishing a categorical boundary between them. In the present study, PMN-MDSC classification was supported by the combination of the viable CD45^+^ CD11b^+^ Ly6G^hi^ Ly6C^lo^ phenotype, pathological expansion after CLP, direct inhibition of T-cell proliferation, and increased Arg-1, ROS, and NO. Ly6G, PD-L1, S100A8/A9, and morphology were not individually regarded as sufficient identity criteria. Morphological analysis could provide additional descriptive information regarding granulocytic maturation, but would not independently determine suppressive activity. Our conclusions therefore concern the splenic expansion, lung-directed migration, and immunosuppressive function of PMN-MDSCs as phenotypically and functionally characterized in this sepsis model.

Several limitations in our experimental design must be acknowledged. First, to minimize mechanistic confounding variables, our study utilized a highly controlled, unresuscitated murine model without the inclusion of broad-spectrum antibiotics or fluid resuscitation. By strictly excluding these standard-of-care interventions, this model serves as an experimental construct specifically designed to isolate fundamental innate immunity and systemic inflammation biology. We acknowledge that this approach inherently diverges from the full complexity of the human clinical condition, where the microbiome and myeloid compartments are profoundly dynamically altered by antibiotics and fluid resuscitation. Consequently, our current in vivo findings should be primarily interpreted as fundamental mechanisms of acute inflammatory biology. Second, our baseline mechanistic studies were primarily conducted in young adult (8 to 10 weeks of age) male mice, despite the profound impact of aging and sexual dimorphism on the host immune response. Adherence to the Minimum Quality Threshold in Pre-Clinical Sepsis Studies (MQTiPPS) guidelines, incorporating aged mice, mixed-sex cohorts, alongside the standardized ICU-like supportive therapies, will be absolutely critical in subsequent translational studies to confirm whether this splenic-pulmonary MDSC circuit is fully conserved across diverse demographic and clinical contexts. Third, while our in vivo knockdown models robustly demonstrate the functional necessity of the TFDP1 axis for PMN-MDSC expansion, a limitation of the current study is the lack of direct promoter occupancy data (e.g., ChIP-seq) to definitively map its downstream transcriptional targets. Future studies will be required to dissect the precise molecular binding landscape of TFDP1 in the septic splenic niche. Furthermore, while our genetic and pharmacological interventions functionally position C1q downstream of PD-1, the precise intracellular signaling cascade linking PD-1 surface activation to C1qa regulation remains to be fully elucidated and represents a critical avenue for our future investigations. Finally, although key AM phenotypes and systemic MDSC expansions were recapitulated in clinical samples, the potential contributions of other extramedullary organs, such as the liver or intestines, to this interorgan circuit warrant further spatiotemporal exploration.

## Materials and Methods

### Animals and ethics statement

Male WT C57BL/6 J mice, *Lyz2*^Cre^ mice, *Pdcd1*^flox/flox^ mice (C57BL/6 background), and CD45.1^+^ congenic mice aged 8 to 10 weeks (body weight: 23.0 to 25.0 g) were obtained from Vital River Laboratory Animal Technology Co Ltd. (Beijing, China). *Lyz2*^Cre^ mice were crossed with *Pdcd1*^flox/flox^ mice to generate *Lyz2*^Cre^*Pdcd1*^flox/flox^ mice. Of note, while the *Lyz2*^Cre^ system mediates robust gene recombination in myeloid lineages, its deletion efficiency in long-lived, tissue-resident macrophages such as AMs is typically high (approximately 75% to 85%) but rarely reaches absolute 100% uniformity. Kaede-tg mice (B6.Cg-Gt(ROSA)26Sor<tm1.1(CAG-kikGR) Kgwa>), a photoconvertible fluorescent protein model, were kindly provided by M. Tomura (Kyoto University). All animals were acclimatized in specific pathogen-free facilities with controlled temperature (22 ± 1 °C), humidity (50% ± 10%), and 12-h light/dark cycles, with ad libitum access to sterilized feed and water. Animal experiments were approved by the Institutional Animal Care and Use Committee of Tongji Medical College (Approval No. 4770) and strictly adhered to NIH guidelines for laboratory animal welfare as well as ARRIVE 2.0 reporting standards.

### “Two-hit” mouse model of sepsis

Polymicrobial sepsis was induced by CLP. Mice were anesthetized with intraperitoneal xylazine (10 mg/kg) and ketamine (100 mg/kg), positioned supine on a heated surgical platform, and prepared under aseptic conditions. After abdominal hair removal and povidone-iodine disinfection, a 1-cm midline incision was made to exteriorize the cecum. The cecum was ligated 1 cm from the tip with 4-0 silk, punctured twice through and through with a 20-gauge needle, and gently compressed to extrude a small droplet of luminal content (approximately 1 mm in diameter) to ensure the patency of the punctures. The cecum was returned to the abdomen, and the muscular and skin layers were closed sequentially with absorbable sutures. For kinetic profiling experiments, the “day 0” (or 0 h) time point designates mice from which tissues were collected immediately following the completion of the CLP procedure, serving as the absolute baseline prior to the systemic progression of sepsis. Conversely, for key mechanistic and interventional comparisons (transcriptomic profiling and epigenetic analyses), time-matched sham-operated control mice were employed. Following surgery, mice were placed on a heating pad until fully recovered from anesthesia. Consistent with our study design to evaluate endogenous migration gradients, no broad-spectrum antibiotics or massive fluid resuscitation were administered.

For the second-hit infection, *P. aeruginosa* strain PAO1 (ATCC 15692) was prepared as follows: Primary cultures were initiated by streaking frozen stocks onto Luria-Bertani (LB) agar plates, followed by overnight incubation at 37 °C. A single colony was selected and incubated in 10 ml of fresh LB broth with continuous agitation (150 rpm) at 37 °C for 16 to 18 h. The overnight culture was then diluted 1:100 into fresh LB broth and grown to the mid-logarithmic phase, reaching an optical density at 600 nm (OD_600_) of approximately 0.5 to 0.6. The bacteria were pelleted by centrifugation (4,000×*g* for 10 min at 4 °C), washed twice with sterile phosphate-buffered saline (PBS), and resuspended. The final bacterial concentration was adjusted to 3 × 10^8^ CFU/ml based on OD_600_ estimations and further confirmed by retrospective serial dilution plating. At 8 d post-CLP (7 d following the initial septic insult), mice were briefly anesthetized with isoflurane inhalation. They subsequently received either PAO1 (5 × 10^6^ CFU) resuspended in 50 μl of sterile PBS or a PBS vehicle control (sham infection) via transoral tracheal instillation under aseptic conditions.

### Pharmacological and genetic interventions

Anti-PD-1 antibody therapy (10 mg/kg, Bioxcell, #BE0273), anti-C1q antibody therapy (5 μg/mouse, GeneTex, #GTX54404), anti-CXCL2 antibody therapy (50 μg/mouse, R&D Systems, #MAB452), or control IgG was delivered intratracheally immediately after CLP.

#### Splenic *Tfdp1*/*S100a9* knockdown

To knock down *Tfdp1* and *S100a9* in the spleen, mice were anesthetized, and a 2-cm subcostal incision was made on the left dorsolateral side to expose the spleen. Using insulin needles, 1 × 10^9^ plaque-forming units (PFU) of Adv-shRNA-*Tfdp1* (#Y38153, Obio), Adv-shRNA-*S100a9* (#Y38156, Obio), or control adenoviruses were injected into the upper, middle, and lower poles of the spleen.

### Splenectomy

To evaluate the functional requirement of the spleen in mediating pulmonary immunosuppression, mice underwent surgical splenectomy or a sham operation 14 d prior to the induction of sepsis. Briefly, mice were anesthetized with intraperitoneal xylazine (10 mg/kg) and ketamine (100 mg/kg) and positioned on a heated surgical platform. The left flank was shaved and prepared under strict aseptic conditions with povidone-iodine. A 1-cm subcostal incision was made through the skin and muscle layers to access the peritoneal cavity. The spleen was carefully exteriorized, and the splenic artery and vein were securely ligated using sterile 4-0 silk sutures before the spleen was completely excised. For sham-operated control mice, the spleen was gently exteriorized and immediately returned to the abdominal cavity without vascular ligation or excision. The muscular layer and skin were sequentially closed with 4-0 absorbable and nonabsorbable sutures, respectively. Mice were allowed to recover on a heating pad and received routine postoperative analgesia. Following a 14-d recovery period to ensure full surgical recuperation and hemodynamic stability, the mice were subjected to the standard CLP procedure.

### Cell culture and treatment

The MH-S murine AM cell line (#CL-0597) was obtained from Punosai Life Science and Technology Co., Ltd. Cells were cultured in Dulbecco’s Modified Eagle Medium supplemented with 10% fetal bovine serum (FBS) and 1% penicillin–streptomycin and maintained at 37 °C in a humidified atmosphere of 5% CO₂/95% air. For stimulation, MH-S cells were treated with LPS (1,000 ng/ml, Sigma-Aldrich, #L2880) and incubated for 24 h before harvesting for analysis. To inhibit Wnt pathway activity, cells were pretreated with iCRT14 (25 μM, Selleck, #S8704) for 4 h before LPS stimulation. To suppress PD-1 expression, MH-S cells were pretreated with an anti-PD-1 antibody (15 μg/ml, Bioxcell, #BE0273) for 6 h prior to LPS stimulation.

### Coculture of macrophages and PD-L1^+^ PMN-MDSCs

Primary murine AMs were isolated from the BALF of mice, and their purity was confirmed by flow cytometry. PD-L1^+^ PMN-MDSCs were isolated from murine spleens via cell sorting 24 h post-CLP. Coculture experiments were performed using either the MH-S cell line or the isolated primary AMs. The macrophages (MH-S cells or primary AMs) were seeded in 6-well plates and stimulated with LPS (1,000 ng/ml; Sigma-Aldrich) in complete media for 6 h. Following this initial stimulation, coculture with PD-L1^+^ PMN-MDSCs was conducted using 2 different methods: (a) Indirect coculture: PD-L1^+^ PMN-MDSCs were placed into Transwell inserts (0.4 μm pore, Corning) at a 10:1 ratio. Specifically, 1 × 10^6^ macrophages were seeded in the lower chamber, and 1 × 10^5^ sorted PD-L1^+^ PMN-MDSCs were seeded in the upper insert. This setup prevents physical cell contact but allows for soluble factor interactions. (b) Direct coculture: Exactly 1 × 10^5^ PD-L1^+^ PMN-MDSCs were added directly into the wells containing 1 × 10^6^ macrophages (maintaining the 10:1 ratio) to enable direct cell-to-cell contact.

### shRNA lentivirus infection

Lentiviruses containing *Klf9* shRNA (Obio, #Y33715) or a negative control sequence were obtained from OBIO Technology (Shanghai, China). MH-S cells were seeded into 6-well plates and infected with the respective shRNA lentivirus at a multiplicity of infection of 60 in complete medium supplemented with 1 μg/μl polybrene. After 12 h, the culture medium was replaced. Transduction efficiency was assessed by fluorescence microscopy, with successful infection defined as ≥80% GFP^+^ cells. Infected cells were either observed for fluorescence expression or harvested for protein extraction and subsequent experiments.

### Phagocytosis assay

MH-S phagocytosis was measured with the Cell Meter Fluorimetric Phagocytosis Assay Kit (Red Fluorescence, #21225). Cells were allowed to attach in 96-well confocal plates and incubated with Protonex 600-labeled beads according to the manufacturer’s protocol. After removal of noninternalized beads by washing, engulfed beads were imaged on a Zeiss LSM 880 confocal microscope. Phagocytic activity was calculated as the percentage of red fluorescent bead area relative to total cell area using ImageJ software (NIH, USA).

### In vitro T-cell suppression assay

To definitively evaluate the immunosuppressive capacity of the mobilized myeloid populations, a CFSE dilution assay was performed. Splenic PMN-MDSCs were isolated from mice 24 h post-CLP via FACS. Responder cells were prepared by mechanically dissociating spleens from naive WT C57BL/6 mice to obtain a single-cell suspension of total splenocytes, followed by erythrocyte lysis. The total splenocytes were washed and labeled with 2.5 μM CFSE in PBS for 10 min at 37 °C in the dark. The labeling reaction was quenched by adding an equal volume of cold complete medium supplemented with 10% FBS, followed by 2 extensive washes. The CFSE-labeled splenocytes were then seeded into 96-well U-bottom plates at a density of 1 × 10^5^ cells per well and stimulated with anti-CD3/CD28 to induce robust T-cell proliferation. Sorted splenic PMN-MDSCs were subsequently added to the corresponding wells at predefined ratios. CFSE-labeled splenocytes cultured with stimulation alone served as the positive proliferation control. After 72 h of coculture at 37 °C in a 5% CO₂ incubator, the cells were harvested, incubated with a fixable viability dye to exclude dead cells, and surface-stained with anti-CD45 and anti-CD3 antibodies. During flow cytometric analysis, viable T cells were specifically identified and gated (Live CD45^+^ CD3^+^), and the proliferation of this specific population, indicated by the stepwise dilution of CFSE fluorescence, was analyzed and quantified utilizing FlowJo software.

### BALF collection and isolation of primary mouse alveolar macrophages

BALF was obtained through a tracheal incision by lavaging the airway 4 times with 500 μl of ice-cold PBS. Cells were collected by centrifugation, and supernatant from the first lavage was saved for cytokine and chemokine assays. Cell pellets were resuspended in PBS and sorted by fluorescence-activated cell sorting (BD FACSAria II, BD Biosciences) to enrich CD45^+^ F4/80^+^ CD11c^+^ mouse AMs. Sorted AMs were used for intervention experiments or protein extraction.

### Parabiosis

Age-matched male C57BL/6 congenic CD45.2 and CD45.1 mice or *Lyz2*^Cre^*Pdcd1*^flox/flox^ and *Pdcd1*^flox/flox^ mice (body weight tolerance: ±0.5 g) were surgically conjoined for parabiosis. Complementary 2-cm dorsolateral skin incisions were made, extending from the olecranon process to the tibial plateau. Articular apposition was achieved by securing the humeral and femoral epiphyses with nonabsorbable 3-0 polypropylene sutures, followed by continuous dermal closure using 5-0 polyglactin absorbable sutures. After surgery, paired mice were housed on clean bedding and received enrofloxacin (0.25 mg/ml) in drinking water. After 4 weeks of vascular equilibration, parabionts underwent CLP. In CD45.1/CD45.2 pairs, lung chimerism was analyzed by flow cytometry 72 h after CLP. Chimerism was calculated as partner-derived CD45.1^+^ or CD45.2^+^ cells divided by total CD45.1^+^ plus CD45.2^+^ cells within each population. In *Lyz2*^Cre^*Pdcd1*^flox/flox^ and *Pdcd1*^flox/flox^ mice, BALF was collected 24 h after the secondary *P. aeruginosa* (PAO1) challenge.

### Adoptive transfer experiments

Single-cell suspensions of splenic PD-L1^+^PMN-MDSCs were isolated via FACS 1 d post-CLP. For adoptive transfer, 2 × 10^5^ highly purified PD-L1^+^PMN-MDSCs were injected intratracheally administered to recipient mice 3 d after CLP surgery.

### Bone marrow cell labeling

Bone marrow cells were labeled through intratibial injection. After exposing the tibia through a small skin incision, 3 μl of Cell-Tracker Deep Red (Thermo Fisher Scientific, C34565) was injected through a needle-drilled opening using a 33-gauge blunt Hamilton needle. Labeling was performed 6 h before CLP. At 0, 24, or 72 h after CLP, bone marrow, spleen, and lung cells were collected and analyzed by flow cytometry for Cell-Tracker Deep Red-labeled PD-L1^+^ PMN-MDSCs.

### Kaede photoconversion

For in vivo tracing of splenic immune cells, spleens of Kaede-tg mice were exposed to a 405-nm laser for 15 min at 24 h after CLP or sham surgery. Adjacent tissues were covered with aluminum foil during illumination to prevent off-target photoconversion.

### Bacterial counting

After terminal anesthesia with xylazine-ketamine, mice underwent thoracotomy and lungs were collected aseptically. Lung tissue was weighed, homogenized in 1 ml sterile PBS, and serially diluted 1:10. Aliquots of 20 μl were plated in triplicate on LB agar and incubated aerobically at 37 °C for 24 h. Colonies were counted and bacterial burden was expressed as CFU per milliliter.

### Western blotting

Proteins were extracted from cells or tissues and quantified with a bicinchoninic acid assay. Equal protein amounts were resolved by sodium dodecyl sulfate–polyacrylamide gel electrophoresis (SDS-PAGE) and transferred to polyvinylidene difluoride (PVDF) membranes (Millipore). Membranes were incubated overnight at 4 °C with antibodies against C1qa (1:1,000, Proteintech, #11602-1-AP), LAG3 (1:1,000, Abclonal, #A2996), CD200 (1:1,000, Abclonal, #A23050), S100A8 (1:1,000, Abclonal, #A15315), S100A9 (1:1,000, Abclonal, #A9842), Wnt5A (1:1,000, Zen Bio, #619919), LEF1 (1:500, Proteintech, #14972-1-AP), KLF9 (1:1,000, Abclonal, #A7196), PD-1 (1:1,000, Abclonal, #A11973), PD-L1 (1:1,000, Abclonal, #A21443), IL-6 (1:1,000, Abcam, #ab229381), TFDP1 (1:1,000, Abclonal, #A5422), IL-10 (1:1,000, Abclonal, #a12255), TGF-β (1:1,000, Abcam, #ab64715), TNF-α (1:1,000, Abclonal, #A11534), PCNA (1:500, Proteintech, #10205-2-AP), or β-actin (1:4,000, Proteintech, #66009-1-Ig). Horseradish peroxidase (HRP)-conjugated anti-mouse or anti-rabbit IgG (AntGene, #ANT019) was used for detection. Bands were visualized by enhanced chemiluminescence (Beyotime), imaged with a UVP gel documentation system, and quantified in ImageJ v1.8.0.

### Flow cytometry analysis

Cell suspensions from lung and spleen were prepared as previously reported [13]. Bone marrow was flushed from femurs and tibiae with ice-cold PBS containing 2% FBS. After erythrocyte lysis, cells were filtered through a 70-μm strainer and washed twice. For staining, 1 × 10^6^ cells were resuspended in 100 μl of 1% BSA/PBS, blocked with anti-CD16/CD32 antibody (5 ng μl^−1^; BD Biosciences, #553141) for 5 min at 4 °C, and incubated with the indicated antibodies. Surface antibodies included fixable viability dye (BD Biosciences, #564406), CD45 (0.5 ng μl^−1^, BioLegend, #103132 or #553079), Ly6C (0.6 ng μl^−1^, BD Biosciences, #560595 or BioLegend, #128032), CD11c (2 ng μl^−1^, BD Biosciences, #564079), PD-L1 (2 ng μl^−1^, BD Biosciences, #564716), PD-1 (2 ng μl^−1^, BD Biosciences, #135219), Gr-1 (2 ng μl^−1^, BD Biosciences, #740850), F4/80 (2 ng μl^−1^, BD Biosciences, #565411), CD206 (5 ng μl^−1^, BioLegend, #141721), CD86 (5 ng μl^−1^, BD Biosciences, #558703), CD11b (2 ng μl^−1^, BD Biosciences, #557960 or #557396), Ly6G (2 ng μl^−1^, BD Biosciences, #560601), CXCR2 (5 ng μl^−1^, BioLegend, #149304), CXCR3 (5 ng μl^−1^, BD Biosciences, #562152), CXCR4 (5 ng μl^−1^, BD Biosciences, #753502), LAG3 (5 ng μl^−1^, BioLegend, #125226), CD45.1 (2 ng μl^−1^, BioLegend, #110736), and CD45.2 (2 ng μl^−1^, BioLegend, #109814). After surface staining, cells were fixed and permeabilized and then stained for IL-10 (5 ng μl^−1^, BioLegend, #505026), CD200 (5 ng μl^−1^, BioLegend, #123810), C1q (5 ng μl^−1^, BD Biosciences, #756671), Ki67 (2 ng μl^−1^, BioLegend, #652403), Arg-1 (5 ng μl^−1^, Invitrogen, #12-3697-82), and Siglec-F (0.5 ng μl^−1^, BD Biosciences, #552125). CXCL2 was detected with anti-CXCL2 primary antibody (4 ng μl^−1^, Proteintech, #98259-1-RR), followed by fluorescent secondary antibody (2 ng μl^−1^, Proteintech, #CL647-98047). Murine PMN-MDSCs were phenotypically enumerated as viable CD45^+^ CD11b^+^ Ly6G^hi^ Ly6C^lo^ cells, whereas M-MDSCs were enumerated as viable CD45^+^ CD11b^+^ Ly6G^-^ Ly6C^hi^ cells. Because the PMN-MDSC phenotype overlaps with that of neutrophils, phenotypic gating alone was not considered sufficient to establish suppressive function. The classification of the CLP-derived populations examined in this study was additionally supported by direct inhibition of T-cell proliferation, together with increased Arg-1 expression and elevated basal ROS and NO production. Phenotype-matched splenic cells from sham mice served as same-tissue steady-state controls. Samples were acquired on a BD LSRFortessa X-20 or Beckman CytoFLEX cytometer and analyzed with FlowJo 10.0.

### Measurement of intracellular ROS and NO levels

Single-cell suspensions prepared from spleens of Sham and CLP (day 3) mice were washed and resuspended in sterile PBS (1 × 10^6^ cells per sample). Unstimulated splenocytes were first incubated with fluorophore-conjugated surface antibodies against CD45, CD11b, Gr-1, Ly6G, and Ly6C, along with a fixable viability dye, for 30 min at 4 °C in the dark to define target cell populations. After washing with cold PBS, cells were resuspended in serum-free RPMI-1640 medium containing either 5 μM DCFDA (Beyotime, #S0035S) for ROS detection or 5 μM DAF-FM DA (Beyotime, #S0019S) for NO detection, and incubated at 37 °C for 30 min in the dark. Following probe loading, cells were washed twice with cold PBS to remove excess free probes. Samples were analyzed immediately without fixation on a Beckman CytoFLEX flow cytometer. Basal ROS and NO levels were quantified by analyzing the MFI using FlowJo software (v10.0).

### Hematoxylin and eosin staining

Lung tissues were fixed in 4% paraformaldehyde (pH 7.4) for 24 h, embedded in paraffin, and cut into 5-μm sections. After deparaffinization in xylene and rehydration through graded ethanol, sections were stained with hematoxylin and eosin (H&E). Slides were dehydrated, mounted, and imaged with an Olympus light microscope. Five randomly selected fields from each section were scored using a validated histopathological injury scale [58].

### Immunofluorescence staining

Lung sections underwent Tyramide Signal Amplification for dual staining with same-species antibodies. Cit-H3 (1:400, Abcam, #ab5103) was probed overnight at 4 °C, followed by HRP-conjugated secondary antibody (30 min, 1:4,000, Abcam, #ab205718) and CY5-tyramide (10 min, dark). After antigen elution (42 °C, 20 min) and blocking (10% goat serum, 37 °C, 30 min), myeloperoxidase (MPO) (1:5,000, Abcam, #ab208670) was labeled with CY3-tyramide. Nuclei were counterstained with 4′,6-diamidino-2-phenylindole.

For MH-S cells, cultures were grown to 40% to 60% confluence on glass coverslips before fixation with 4% paraformaldehyde (20 min, RT). Following permeabilization with 0.2% Triton X-100 (5 min) and blocking with 10% goat serum (37 °C, 30 min), sequential staining was performed for PD-1 (1:200, Abcam, #ab214421, overnight at 4 °C; detected with FITC-tyramide), F4/80 (1:400, Cell Signaling Technology, #70076; CY3-tyramide), and KLF9 (1:100, Santa Cruz, SC-376422; CY5-tyramide), with antigen retrieval and blocking steps repeated between each primary antibody. All samples were imaged using a Case Viewer whole-slide scanner (3D Histech) and analyzed with ImageJ software (v1.8.0, NIH).

### Determination of cytokine levels

Mouse BALF cytokines, including IL-6, IL-17A, TNF-α, and IFN-γ, were measured with the BD mouse Th1/Th2/Th17 cytokine cytometric bead array kit (#560485) following the manufacturer’s protocol. Chemokines were measured in lung homogenate supernatants using ELISA kits for CXCL1 (BOSTER, #EK0723), CXCL2 (BOSTER, #EK0452), and CXCL5 (BOSTER, #EK0919).

### Co-immunoprecipitation

MH-S cells and mouse lung tissues were lysed for 30 min on ice in NP-40 lysis buffer (1% NP-40, 150 mM NaCl, and 50 mM Tris-HCl, pH 7.4) containing protease inhibitors. After centrifugation, clarified lysates were incubated overnight at 4 °C with anti-PD-1 antibody for MH-S cells (1:50, Cell Signaling Technology, #84651) or anti-LEF1 antibody for lung tissue (1:50, Proteintech, #14972-1-AP). Antibody–protein complexes were captured with Protein A/G magnetic beads, washed extensively, eluted in sample buffer, separated by SDS-PAGE, and immunoblotted for KLF9 or C1qa using the corresponding antibodies.

### Dual-luciferase reporter assay

To validate direct transcriptional regulation, 293T cells were seeded into 96-well plates at a density of 1.5 × 10^4^ cells per well and cultured overnight. Cells were then cotransfected with specific transcription factor overexpression plasmids (LEF1 or KLF9) alongside their corresponding WT or mutated (Mut) promoter reporter plasmids (*Klf9* or *Pdcd1*). Transfections were performed using GCTrans-siRNA/DNA transfection reagent in OPTI-MEM according to standard protocols. At 36 to 48 h post-transfection, the culture medium was discarded, and cells were washed with PBS before being lysed with 50 μl of lysis buffer for 10 min. The lysates were centrifuged at 10,000×*g* for 5 min, and 20 μl of the clarified supernatant was transferred to an assay plate. Firefly (FLuc) and Renilla (RLuc) luciferase activities were sequentially quantified using a Synergy 2 microplate reader (BioTek) following the addition of 100 μl of their respective specific substrates. The relative transcriptional activity for each group was calculated as the ratio of Firefly luciferase luminescence to Renilla luciferase luminescence (FLuc/RLuc) to correct for transfection efficiency variations.

### Patient population and isolation of human alveolar macrophages

Twelve adult ICU patients from Wuhan Union Hospital were enrolled, including 6 patients with sepsis diagnosed within 24 h of ICU admission according to Sepsis-3 criteria and 6 nonsepsis controls. The study was approved by the Ethics Committee of Huazhong University of Science and Technology (2023-0143-01), and informed consent was obtained. BALF was collected by bronchoscopy under topical 1% lidocaine anesthesia. Three aliquots of 20 to 30 ml of sterile saline were instilled into the right middle lobe or lingular segments. BALF was filtered through a 40-μm strainer, centrifuged at 420×*g* for 10 min at 4 °C, and washed twice with PBS. Cells were stained with fixable viability dye (BD Biosciences, #564406), CD45 (0.5 ng μl^−1^, BioLegend, #304027), CD169 (0.5 ng μl^−1^, BioLegend, #307609), and HLA-DR (0.5 ng μl^−1^, BioLegend, #361611) and sorted to enrich CD45^+^ CD11b^+^ HLA-DR^+^ CD169^+^ human AMs.

### Flow cytometric analysis of clinical peripheral blood

To evaluate the systemic mobilization of PMN-MDSCs, 2 ml of EDTA-anticoagulated arterial blood was collected from 6 septic patients and 6 nonseptic ICU controls within 72 h of sepsis diagnosis or ICU admission. Peripheral blood mononuclear cells were isolated using a commercial lymphocyte separation medium via standard density gradient centrifugation. Following isolation, the cells were washed twice with cold PBS and resuspended in FACS buffer. For flow cytometric analysis, the cell suspensions were first incubated with human Fc receptor blocking solution and stained with a fixable viability dye to exclude dead cells. Subsequently, the cells were surface-stained with a panel of human-specific antibodies, including CD45 (0.5 ng μl^−1^, BD Biosciences, # 557833), CD11b (0.5 ng μl^−1^, BD Biosciences, # 557396), CD15 (0.5 ng μl^−1^, BD Biosciences, # 755956), CD14 (0.5 ng μl^−1^, BD Biosciences, # 555399), CD33 (0.5 ng μl^−1^, BD Biosciences, # 740400), HLA-DR (0.5 ng μl^−1^, BD Biosciences, # 757334), CXCR2 (0.5 ng μl^−1^, BD Biosciences, # 555933), and PD-L1 (1 ng μl^−1^, BD Biosciences, # 563738). Data were acquired on a flow cytometer and analyzed using FlowJo software. Human PMN-MDSCs were strictly defined as viable CD45^+^ CD11b^+^ CD15^+^ CD14^−^ CD33^+^ HLA-DR^l^°^w^ cells, and the frequencies of the CXCR2^+^ PD-L1^+^ subpopulations within this gate were further quantified.

### ChIP-seq analysis

For ChIP-seq, MH-S cells were grown in 15-cm dishes to approximately 60% confluence and harvested after 24 h. DNA was purified according to the manufacturer’s instructions, and sequencing libraries were prepared with the ThruPLEX DNA-seq kit. Libraries of 200 to 500 bp were enriched, quantified, and sequenced on an Illumina HiSeq 2500. Anti-LEF1 antibody from Cell Signaling Technology was used for chromatin immunoprecipitation. ChIP-seq processing was performed by Gene Read (Wuhan, China).

### RNA-seq analysis

RNA-seq for MH-S cells was performed by Biotree Biotech. In brief, MH-S cells were divided into 3 experimental groups (*n* = 3/group): (a) coculture with PD-L1^+^ PMN-MDSCs + LPS (1,000 ng/ml), (b) LPS alone (1,000 ng/ml), and (c) PBS control. Total RNA was extracted using Trizol Reagent (Thermo Fisher Scientific) and quantified with an ND-1000 spectrophotometer (NanoDrop Technologies, Inc., Wilmington, DE, USA). RNA quality was assessed using a Bioanalyzer 2100 (Agilent Technologies, Inc.) with an RNA 6000 LabChip kit. Libraries were sequenced on an Illumina NovaSeq 6000 platform with 150-bp paired-end reads. Differentially expressed genes were identified with edgeR in R using fold change >2.0 and *P* < 0.05. GO and Kyoto Encyclopedia of Genes and Genomes (KEGG) analyses were used for functional enrichment.

### Integrative analysis of bulk RNA-seq and ATAC-seq

RNA-seq and ATAC-seq of splenic PD-L1^+^ PMN-MDSCs were performed by Seqhealth Technology Co. Ltd. (Wuhan, China). Cells were sorted from spleens 24 h after CLP or sham surgery (*n* = 3 per group). Total RNA was extracted, assessed for quality, converted into sequencing libraries, and sequenced on an Illumina NovaSeq 6000 platform. Differentially expressed genes were selected using fold change >2.0 and *P* < 0.05.

For ATAC-seq, 500,000 sorted cells were used for nuclei preparation. Cells were washed with cold PBS, resuspended in cold lysis buffer, and centrifuged at 500×*g* for 10 min at 4 °C. After the removal of the supernatant, nuclei were resuspended in nuclease-free water. Transposition and library construction were performed with the TruePrep DNA Library Prep Kit V2 for Illumina (TD501; Vazyme Biotech). Libraries were enriched, quantified, and sequenced on an Illumina NovaSeq 6000 (Illumina, San Diego, CA, USA).

For integrated bulk RNA-seq and ATAC-seq analysis, up-regulated and down-regulated DEGs (*P*_adj_ < 0.05 and |log2FC| > 1) were compared with open and closed differentially accessible regions from ATAC-seq (*P*_adj_ < 0.05 and |log2FC| > 1).

### Single-cell sequencing data analysis

#### Single-cell suspension preparation

Lungs were harvested from euthanized mice, minced, and digested in RPMI-1640 containing FBS, collagenase IV (0.1 mg/ml), and DNase I (0.1 mg/ml) at 37 °C for 40 min. The digested tissue was converted into a single-cell suspension, filtered through a 40-μm strainer, and centrifuged at 500×*g* for 5 min at 4 °C. Red blood cells were lysed before downstream processing. scRNA-seq analyses were completed with DNBseq platform (BGI, Wuhan Technology Center, China).

#### Single-cell RNA sequencing

Single-cell libraries were prepared with the DNBelab C Series High-throughput Single-cell RNA Library Preparation Set V3.0 (MGI, China). Single-cell suspension, oil, and beads were loaded sequentially into the C4 scRNA slide and instrument. After droplet generation, gel beads-in-emulsion (GEMs) were collected for reverse transcription and cDNA amplification. Indexed libraries were constructed according to the manufacturer’s protocol and sequenced on the appropriate high-throughput platform.

#### Unsupervised cell clustering

Unsupervised analysis was performed with Seurat v3.2 in R v4.0.3. Genes detected in fewer than 5 cells were removed before analysis. Cells were filtered by gene number, unique molecular identifier (UMI) count, and mitochondrial transcript fraction. After normalization and variable feature selection, datasets were scaled, subjected to dimensionality reduction, clustered, and analyzed for marker genes.

#### Cell-type annotation

Seurat (version 5.3.0) was used to perform clustering analysis on the single-cell dataset. The data was read into R (version 4.5.1) as a count matrix and scaled with a size factor of 10,000 followed by log transformation. Cells were filtered using the criteria “nFeature_RNA > 200 & nFeature_RNA < 6000 & percent.mt < 10”. The single-cell data were integrated using the “integrated.cca” function. For Uniform Manifold Approximation and Projection (UMAP) and clustering analysis, the top 30 principal components were used. The “FindAllMarkers” function was employed to identify specific markers for each cluster recognized by Seurat. Automated cell-type assignment was performed with CellMarkerAccordion (version 1.0.0). Briefly, the top 30 marker genes per cluster were queried against the Mouse-spleen compendium. For each cluster, the algorithm returned a ranked list of putative cell identities together with area under the receiver operating characteristic (AUROC)-based confidence scores and the contributing marker genes. Clusters whose top hit exhibited AUROC > 0.85 and whose marker expression pattern agreed with canonical literature were directly adopted. Final cluster identities were determined by integrating CellMarkerAccordion predictions with manual curation.

#### MDSC score

Module scores for the MDSC gene sets were calculated with AddModuleScore from the Seurat package. Four MDSC signatures (Table S1) were compiled from the literature: 2 mouse (M.MDSC) sets [59,60] and 2 human (H.MDSC) sets [61,62] that were converted to mouse orthologues for extension and reference. The gene sets for MDSC subset function scores were referenced from the data compiled by Xie et al. [63].

#### Enrichment of neutrophil-associated signaling pathways

Mouse neutrophil-related gene sets (*n* = 51) were retrieved from the GO and Reactome databases via the SearchDatabase function of the SeuratExtend package (version 1.2.5). Per-cell signature activity was quantified with AUCell, and the resulting AUC matrix was stored in @misc$AUCell$genesets. For each Seurat cluster, *z*-scores were computed for the top enriched gene sets (ranked by *P* value) and displayed as a heatmap.

#### Pseudotime trajectory analysis

We conducted pseudotime trajectory analysis on the scRNA-seq dataset using the Monocle2 (version 2.34.0) package to explore dynamic gene expression patterns across different cell states. During data preparation, cell identities were assigned based on Seurat clustering, and raw gene expression data were retrieved and converted into a sparse matrix for Monocle compatibility. Metadata and gene annotations were also formatted for downstream analysis. Subsequently, a Monocle object (cds) was created using the processed data. In the data preprocessing step, normalization was performed by estimating size factors and dispersions, and cells with low gene expression as well as genes expressed in fewer than 10 cells were filtered out. For trajectory analysis, trajectory genes were selected, dimensionality reduction was carried out using the DDRTree method, and cells were ordered along the pseudotime trajectory. Finally, the pseudotime trajectory was visualized by cell type, pseudotime, and experimental condition, and gene expression patterns along the trajectory were analyzed.

The single-cell sequencing data for Fig. 3 was sourced from the NCBI Gene Expression Omnibus (GEO) database (GSE207651). In our research, clusters were annotated according to the CellMarker database and marker genes of specific cell type reported in research. Specifically, AMs were identified using the following markers: *Itgax*, *Itgam*, *Adgre1*, and *Siglecf*.

### Statistical analysis

Data are shown as mean ± SD and were analyzed with GraphPad Prism 9. Heatmaps and correlation plots were generated with ChiPlot (https://www.chiplot.online/, accessed on 2026 June 6). Group comparisons were performed using Student *t* test or one-way analysis of variance (ANOVA) with Tukey’s multiple-comparison test as appropriate. Survival curves were compared with the log-rank (Mantel–Cox) test. *P* < 0.05 was considered statistically significant.

## Data Availability

All data are available in the main manuscript or the Supplementary Materials. Address correspondence to: Y.S. (you_shanghust@163.com) and J.Z. (zhjcheng1@126.com).
