# Supplementary material for: Splenic PD-L1+ Myeloid-Derived Suppressor Cells Induce Contact-Dependent Alveolar Macrophage Dysfunction in Early Sepsis
Source: Research (Wash D C). 2026 Aug 19;9:1407. doi: 10.34133/research.1407 (PMC13486733; doi:10.34133/research.1407)
Supplement: Supplementary file 1 [file research.1407.f1.docx]

**SUPPLEMENTARY MATERIALS**

**Figures S1 to S8**

**Figure S1. Single-cell transcriptomic characterization of spleen-derived immune cells in the lung.**

(**A**) Dot plot displaying the top marker genes used for annotating the major cell types identified among all spleen-derived (Kaede-red^+^) immune cells. (**B**) Dot plot of the top marker genes for subclusters within the re-clustered myeloid cell compartment. (**C**) Single-cell clustering and cell type annotated results of re-clustered MDSCs. (**D**) Functional scores for key biological processes across the MDSC subclusters. (**E**) Expression pattern of genes associated with immunosuppressive functions in MDSC subclusters. (**F**) Expression pattern of canonical MDSC marker genes across the identified subclusters. (**G**) Pseudotime trajectory analysis of MDSCs, colored by subcluster, illustrating inferred developmental relationships. (**H**) Pseudotime trajectory analysis of MDSCs, colored by sample group (time post-CLP), showing the temporal distribution of cells along the differentiation trajectory.

**Figure S2. Wnt signaling activation upregulates PD-1 via KLF9.**

(**A**) Protein levels of β-catenin and LEF1 in AMs (n=6). (**B**) Promoter-specific binding peaks of Lef1 at the KLF9 locus. (**C**) KLF9 binding motif (JASPAR). (**D**) Protein levels of KLF9 and PD-1 in AMs (n=7). (**E**) Correlation analysis between PD-1 and KLF9 expression (n=7). (**F**) Co-immunoprecipitation and western blot showing PD-1–KLF9 interaction in AMs. (**G**) Fluorescence staining of KLF9 lentiviral transfection in AMs. (**H**) Western blot and quantification of KLF9 and PD-1 in AMs (n=6). (**I**) Relative luciferase activity of the wild-type (WT) or mutant (Mut) Pdcd1 promoter reporters co-transfected with a KLF9 overexpression vector (KLF9-OE) or negative control (OE-NC) in 293T cells (n = 4). (**J**) Relative luciferase activity of the wild-type (WT) or mutant (Mut) Klf9 promoter reporters co-transfected with an LEF1 overexpression vector (LEF1-OE) or negative control (OE-NC) in 293T cells (n = 4). Values are shown as mean ± SD; Statistical testing for panel (A, D, I and J) was determined using one-way ANOVA with Tukey’s multiple-comparison test; for panel (H), Student’s *t* test was used. **P* < 0.05, ***P* < 0.01, ****P* < 0.001, *****P* < 0.0001.

**Figure S3. PD-L1⁺ PMN-MDSCs exert immunomodulatory effects on AMs.**

(**A**) Schematic of CLP model, tissue harvesting and processing. (**B**) Flow cytometric gating strategy for viable T cells (Live CD45⁺ CD3⁺), representative CFSE dilution histograms, and quantification of the T cell proliferation percentage after 3 days of co-culture with vehicle or sorted splenic PMN-MDSCs (n=5). (**C**) Representative flow cytometry histograms and statistical quantification of intracellular Arginase-1 (Arg-1) expression in Live CD45⁺ CD11b⁺ Gr-1⁺ Ly6Gʰⁱ Ly6Cˡᵒʷ splenic cells from Sham (classical neutrophils) and CLP 3d mice (PMN-MDSCs) (n=6). (**D, E**) Representative flow cytometry fluorescence peak shifts and mean fluorescence intensity (MFI) quantification of basal ROS (DCFDA probe) and NO (DAF-FM DA probe) production (n=6). (**F**) Western blot and quantification of PD-1 in AMs (n=5), and PD-L1 in PMN-MDSCs (n=5). (**G**) Schematic of co-culture setup. (**H**) Representative fluorescent images of KLF9 and PD-1 expression AMs. Scale bar = 10 μm. (**I** to **K**) FCM analysis using t-SNE algorithm showing expressions of PD-1, IL-10, M1 and M2 polarization in AMs (I), percentage of PD-1 (n=10) (J), IL-10 (n=10) (K), in AMs. (**L** to **O**) Representative FCM plots (L), percentages of CD86 (n=10) (M), CD206 (n=10) (N) and M1/M2 ratio (n=10) (O), in AMs. (**P**) Heat map illustrating the correlations between PD-1, IL-10, CD86, CD206, and M1/M2 ratio. (**Q**) Kaplan-Meier survival analysis following *Pseudomonas aeruginosa* infection (n=20). (**R**) Representative H&E sections of lung, immune cells marked by red arrows. (**S**) Representative fluorescent images of neutrophils in lung. Scale bar = 50 μm. (**T**) BALF levels of IL-6, IL-17A and TNF-α in mice (n=9 to 10). Data were plotted as mean ± SD for each data point; Statistical significance for panels (B to E, J, K, M to O, and T), Student’s *t* test was used; Statistical significance for panel (F) was determined using one-way ANOVA followed by Tukey’s multiple-comparison test; for panel (Q), *P* values were calculated using the Log-rank (Mantel-Cox) test; **P* < 0.05, ***P* < 0.01, ****P* < 0.001, *****P* < 0.0001. ns, not significant.

**Figure S4. The immunosuppressive influence of PD-L1⁺ PMN-MDSCs on AMs is contact-dependent.**

(**A** and **B**) Example FCM plots and percentages of PD-1 (n=6 to 7) (A), and IL-10 (B) (n=10) in LPS-stimulated MH-S cells co-cultured with or without Transwell-separated PD-L1⁺ PMN-MDSCs. (**C** to **F**) Example FCM plots (C) and percentages of CD86 (n=6 to 7) (D), CD206 (E) (n=6 to 7), and M1/M2 ratio in LPS-stimulated MH-S cells co-cultured with Transwell-separated PD-L1⁺ PMN-MDSCs or not. (**G**) Representative phagocytosis assay images and quantification in MH-S cells directly co-cultured with PD-L1⁺ PMN-MDSCs (n = 6). (**H**) Expression of the inflammatory factors IL-6 and TNF-α in cellular supernatant. (**I**) Flow cytometric validation of the purity of primary murine alveolar macrophages (AMs) isolated from bronchoalveolar lavage fluid (BALF). (**J** to **M**) Representative flow cytometry plots (J, K) and corresponding statistical quantification (L, M) of PD-1, IL-10, and C1q expression in LPS-stimulated primary AMs co-cultured with or without PD-L1⁺ PMN-MDSCs under direct contact (J, L) or Transwell-separated indirect contact (K, M) conditions. Values are shown as mean ± SD; Statistical testing for panel (A, B, D to F, L and M) was determined using Student’s *t*-test; for panel (G and H), one-way ANOVA with Tukey’s multiple-comparison test was used. ***P* < 0.01, *****P* < 0.0001. ns, not significant.

**Figure S5. PD-1 expression drives AM exhaustion in vitro.**

(**A** to **F**) Example FCM plots (A to C), and percentages of Lag3 (n=7) (D), C1q (n=7) (E), CD200 (n=7) (F) in LPS-stimulated AMs co-cultured with PD-L1+PMN-MDSCs. (**G** to **L**) Example FCM plots (G, H, I), and percentages of Lag3 (n=6) (J), C1q (n=6) (K), CD200 (n=6) (L) in AMs with high versus low PD-1 expression co-cultured or not. (**M**) Quantification of LPS-stimulated AMs co-cultured with PD-L1⁺ PMN-MDSCs (n=6). Values are shown as mean ± SD; Statistical testing was determined using Student’s *t-*test. **P* < 0.05, ***P* < 0.01, ****P* < 0.001, *****P* < 0.0001. ns, not significant.

**Figure S6. PD-1 expression drives AM exhaustion in vivo.**

(**A**) Flow cytometric gating strategy to group AMs by PD-1 level. (**B** to **G**) Example FCM plots (B to D), and percentages of Lag3 (n=8) (E), C1q (n=8) (F), CD200 (n=8) (G) in AMs of septic lungs. (**H**) The quantification of AMs of septic lungs (n=3 to 4). Values are shown as mean ± SD; Statistical testing for panel (E to G) was determined using Student’s *t*-test; for panel (H), one-way ANOVA with Tukey’s multiple-comparison test was used. **P* < 0.05, ***P* < 0.01, ****P* < 0.001. ns, not significant.

**Figure S7. Anti–PD-1 improves outcomes after polymicrobial sepsis and secondary *Pseudomonas aeruginosa* challenge.**

(**A**) Example FCM plots and percentage of Ki67 in AMs (n=4). (**B**) Protein levels of PD-1, C1qa, Lag3, CD200, IL-10, TGF-β, TNF-α and IL-6 in AMs. (**C**) Representative FCM histograms and percentage of expression of PD-1 in flow-sorted AMs from murine lungs (n=8). (**D** to **H**) Representative FCM histograms (D), quantification of AMs (E), and percentages of C1q (F), Lag3 (G), and CD200 (H) in AMs (n=8). (**I**) Kaplan-Meier analysis of survival of septic mice after Pseudomonas aeruginosa infection (n=17 to 18). (**J**) Representative H&E sections of lung. (**K**) BALF levels of IL-6, TNF-α and IFN-γ in septic mice. (**L**) Representative fluorescent images of NETs formation in the lung. Scale bar = 50 μm. Values are shown as mean ± SD; Statistical testing for panel (A) was determined using one-way ANOVA with Tukey’s multiple-comparison test; for panel (C, E to H, and K), Student’s *t*-test was used; for panel (I), *P* values were calculated using the Log-rank (Mantel-Cox) test. **P* < 0.05, ***P* < 0.01, ****P* < 0.001, *****P* < 0.0001.

**Figure S8. C1q blockade improves outcomes after polymicrobial sepsis and secondary *Pseudomonas aeruginosa* challenge.**

(**A**) Schematic of CLP modeling and anti-C1q antibody administration. (**B**) Kaplan-Meier analysis of survival of septic mice after *Pseudomonas aeruginosa* infection (n=19 to 23). (**C**) BALF levels of IL-6, IFN-γ and TNF-α one day after *Pseudomonas aeruginosa* infection. (**D**) Western blot and quantification of C1qa, PD-1, Lag3, CD200, IL-10, TGF-β in flow-sorted AMs (n=6). (**E**) Co-immunoprecipitation and western blot showing LEF1–C1qa interaction in AMs. (**F**) Western blot and quantification of C1qa in LPS-treated AMs administered with iCRT14 or vehicle (n=6). Data were plotted as mean ± SD for each data point; Statistical significance for panel (B) was determined using the Log-rank (Mantel-Cox) test.; for panel (C), Mann-Whitney test was used; for panel (D and F), Student’s *t* test was used. **P* < 0.05, *****P* < 0.0001. ns, not significant.

**Supplementary Tables**

**Table S1: MDSC signature genes from top two gene sets**

| PMID: 32086381 | PMID: 40107423 | PMID: 38217732 | PMID: 40381622 |
| --- | --- | --- | --- |
| M.MDSC.1 | M.MDSC.2 | H.MDSC.1 | H.MDSC.2 |
| *Wfdc17* | *S100a9* | *ARHGAP26* | *CD14* |
| *Ifitm1* | *S100a8* | *ATP2B1* | *FUT4* |
| *Il1b* | *Cd84* | *B4GALT5* | *ITGAM* |
| *BC100530* | *Tgfb1* | *CD53* | *CD33* |
| *Srgn* | *Lcn2* | *CDKN1A* | *CEACAM8* |
| *Gm5483* | *Cebpb* | *CHD1* | *S100A9* |
| *Stfa2l1* | *Arg1* | *CREM* | *S100A8* |
| *Stfa2* | *Adam17* | *CSRNP1* | *ARG1* |
| *Prok2* | *Lgals9* | *CXCL2* | *IDO1* |
| *Ifitm2* | *Hif1a* | *CXCR4* | *NOS2* |
| *Junb* | *Hmox1* | *CYBB* | *NCF1* |
| *Dusp1* | *Stat3* | *CCR1* | *PROK2* |
| *Socs3* | *Vegfa* | *CCL3L1* | *IL4R* |
| *Btg1* | *Nos1* | *CCL3* |  |
| *1600014C10Rik* | *Olr1* | *CASS4* |  |
| *Selplg* | *Il4ra* | *DSE* |  |
| *Asprv1* | *Cd274* | *DUSP2* |  |
| *Igfbp6* | *Nos2* | *EFHD2* |  |
| *Gm5150* | *Ptgs2* | *EMILIN2* |  |
| *Pla2g7* | *Il10* | *FCER1G* |  |
| *Csf3r* | *Il6* | *FCGR1A* |  |
| *Cxcr2* |  | *FNDC3B* |  |
| *Tpd52* |  | *FOSL2* |  |
| *Tspo* |  | *FTH1* |  |
| *Cyp4f18* |  | *GK* |  |
| *Grina* |  | *GRINA* |  |
| *Fabp5* |  | *GSTO1* |  |
| *Clec4d* |  | *HBEGF* |  |
| *Steap4* |  | *HCAR2* |  |
| *Map1lc3b* |  | *HIF1A* |  |
| *Ccr1* |  | *HIP1* |  |
| *Lrg1* |  | *HLA-B* |  |
| *Clec4e* |  | *ICAM1* |  |
| *Ctsd* |  | *IER3* |  |
| *Cd84* |  | *IFITM3* |  |
| *Gcnt2* |  | *IL1B* |  |
| *Arg2* |  | *INAFM2* |  |
| *BC117090* |  | *LAT2* |  |
| *Npl* |  | *LCP2* |  |
| *Fgl2* |  | *LIMK2* |  |
| *Rnf149* |  | *LRRFIP1* |  |
| *Sephs2* |  | *LYST* |  |
| *S100a6* |  | *MAN1A2* |  |
| *Lmnb1* |  | *MAP3K8* |  |
| *Amica1* |  | *MARCKSL1* |  |
| *Eif4ebp1* |  | *METRNL* |  |
| *Msrb1* |  | *NFKBIA* |  |
| *Ubb* |  | *NLRP3* |  |
| *C5ar1* |  | *OLR1* |  |
| *Ypel3* |  | *PECAM1* |  |
| *Fcgr3* |  | *PIM3* |  |
| *Gsr* |  | *PLSCR1* |  |
| *Taldo1* |  | *PNRC1* |  |
| *Atp6v1g1* |  | *PPIF* |  |
| *S100a11* |  | *PTAFR* |  |
| *Hp* |  | *RHOQ* |  |
| *Alox5ap* |  | *RIPK2* |  |
| *Litaf* |  | *RTN4* |  |
| *Txn1* |  | *SBNO2* |  |
| *Upp1* |  | *SDCBP* |  |
| *AB124611* |  | *SECTM1* |  |
| *Gpcpd1* |  | *SERPINB9* |  |
| *Snap23* |  | *SIGLEC10* |  |
| *Il4ra* |  | *SPHK1* |  |
| *Hist1h2bc* |  | *SLA* |  |
| *Retnlg* |  | *STXBP2* |  |
| *Myd88* |  | *TLR2* |  |
| *Adipor1* |  | *TNFAIP2* |  |
| *Stk17b* |  | *TNFAIP3* |  |
| *Zyx* |  | *YPEL5* |  |
| *Rgs3* |  | *ZSWIM6* |  |
| *Il1f9* |  |  |  |
| *Hdc* |  |  |  |
| *Atg3* |  |  |  |
| *Gda* |  |  |  |
| *Slc40a1* |  |  |  |
| *Tarm1* |  |  |  |
| *Cdk2ap2* |  |  |  |
| *Stfa3* |  |  |  |
| *Glipr2* |  |  |  |
| *Tacstd2* |  |  |  |
| *Picalm* |  |  |  |
| *Mtus1* |  |  |  |
| *Fbxl5* |  |  |  |
| *Slfn1* |  |  |  |
| *Rnd1* |  |  |  |
| *Ier2* |  |  |  |
| *Mxd1* |  |  |  |
| *Siglece* |  |  |  |
| *Cdkn2d* |  |  |  |
| *Cd33* |  |  |  |
| *Lilr4b* |  |  |  |
| *Cd14* |  |  |  |
| *Sell* |  |  |  |
| *Ppt1* |  |  |  |
| *Skap2* |  |  |  |
| *Sfxn5* |  |  |  |
| *2810474O19Rik* |  |  |  |
| *Ikbkap* |  |  |  |
| *Hbb-bs* |  |  |  |
| *Atp11b* |  |  |  |
| *Cxcl2* |  |  |  |
| *Osm* |  |  |  |
| *Ier3* |  |  |  |
| *Hba-a1* |  |  |  |
